# 18KHT01, a Potent Anti-Obesity Polyherbal Formulation

**DOI:** 10.3389/fphar.2021.807081

**Published:** 2021-12-17

**Authors:** Prakash Raj Pandeya, Ramakanta Lamichhane, Gopal Lamichhane, Kyung-Hee Lee, Hyeong Kyu Lee, Su-jin Rhee, Hyun-Ju Jung

**Affiliations:** ^1^ Department of Oriental Pharmacy and Wonkwang-Oriental Medicines Research Institute, Wonkwang University, Iksan, South Korea; ^2^ Bio-Safety Research Institute, Jeonbuk National University, Iksan, South Korea; ^3^ Natural Medicine Research Center, Korea Research Institute of Bioscience and Biotechnology (KRIBB), Cheongju, South Korea; ^4^Department of Pharmacy, Wonkwang University, Iksan, South Korea

**Keywords:** 18KHT01, green tea, polyherbal formulation, anti-obesity activity, diet-induced obesity (DIO, ), synergy evaluation

## Abstract

Obesity is a life-threatening metabolic disorder necessitating urgent development of safe and effective therapy. Currently, limited such therapeutic measures are available for obesity. The present study was designed to develop a novel, safe and effective herbal therapy for the management of obesity. A polyherbal formulation (18KHT01) was developed by homogeneously mixing a specific proportion of crude *Quercus acutissima* (acorn jelly powder), *Camellia sinensis* (dry leaf buds), and *Geranium thunbergii* (dry aerial part) along with *Citrus limon* (fruit juice). Synergistic antioxidant, antiadipogenic, and anti-obesity activities were evaluated by *in vitro* as well as *in vivo* studies. *In vitro* experiments revealed strong synergistic antioxidant and anti-adipogenic activities of 18KHT01. Molecular assessment of 18KHT01 showed significant down-regulation of vital adipogenic factors such as PPARγ, C/EBPα, aP2, SREBP-1c, FAS, and LPL. Based on the results of the preliminary toxicity study, 75 and 150 mg/kg, twice daily doses of 18KHT01 were administered to evaluate anti-obesity activity in diet-induced obese (DIO) C57BL/6J mice model. The major obesity-related parameters such as body weight, weight gain, food efficiency ratio, as well as serum lipid profile were significantly reduced by 18KHT01 with potential synergism. Also, the high-fat diet-induced insulin resistance was suggestively alleviated by the formulation, and thus ameliorated fasting blood glucose. Histological evaluation of liver and white adipose tissue revealed that the significant reduction of fat depositions and thus reduction of these tissue weights. Synergy evaluation experiments exhibited that the 18KHT01 offered strong synergism by improving efficacy and reducing the toxicity of its ingredients. Overall results evidenced the 18KHT01 as a safe and potent anti-obesity herbal therapy.

## Introduction

Obesity is considered as the accumulation of abnormal or excessive fat into the body to the extent that may impair health conditions. According to World Health Organization (WHO), more than 1.9 billion adults were overweight and 650 million were suffering from obesity, which includes about 40% of the world’s population and is still in an increasing pattern (WHO, 2021a; WHO, 2021b). Obesity considerably increases the risk of several metabolic diseases such as type 2 diabetes mellitus, hypertension, stroke, fatty liver disease, myocardial infarction, obstructive sleep apnea, and several cancers (Bluher, 2019). Adipogenesis, a metabolic onset of obesity, is a complex process where pre-adipocytes get differentiated into adipocytes. The differentiated adipose cells in white adipose tissue exhibit various morphological and biochemical modifications such as triglyceride accumulation, insulin-regulated metabolism, and expression and regulation of various adipogenic factors such as PPARγ, C/EBPα, SREBP-1c, LPL, FAS, and FABP4 (Gregoire, 2001; Sarjeant and Stephens, 2012). Primarily, obese people are recommended to modify their lifestyle by increasing physical activity and reducing calories intake for the treatment of obesity. When these behavioral changes do not give significant results, pharmacologic treatment is required. But, most anti-obesity drugs have serious adverse effects. Phentermine, diethylpropion, mazindol, rimonabant, fenfluramine, and dexfenfluramine have a current history of withdrawing due to having serious adverse effects (Glazer, 2001; Kang and Park, 2012). Thus, it is essential to develop safe and effective natural medication for the treatment of obesity. Evidences are arising to support the effectiveness of herbal medicine and polyherbal formulations in the treatment of obesity. Yun (2010) and Liu et al. (2017) have reviewed anti-obesity herbal medicines and explored their possible mechanism of action (Yun, 2010; Liu et al., 2017). Generally, herbal medicines exhibited anti-obesity activity through lipase inhibition, food intake suppression, increase of energy expenditure (thermogenesis), adipogenesis inhibition, lipid metabolism regulation, insulin secretion stimulation, increase of satiety, diuresis stimulation, and by acting on the central nervous system through leptin (Saper et al., 2004; Yun, 2010; Chandrasekaran et al., 2012; Liu et al., 2017). A combination of several medicinal herbs to achieve additional therapeutic effectiveness is stated as polyherbalism. This pharmaceutical approach helps in bringing increased therapeutic effects and reduced adverse effects (Parasuraman et al., 2014; Bisht and Ram, 2017). The different components of a polyherbal formulation may exert synergistic, potentiative, agonist, additive, and antagonistic actions through diverse active principles, thus displaying a dynamic way to exhibit wide therapeutic range (Benzie and Wachtel-Galor, 2011; Karole et al., 2019). The positive interactions among herbal ingredients in a polyherbal formulation may be either synergism or additive (Zhou et al., 2016). In this study, an anti-obesity polyherbal formulation (18KHT01) was developed by homogeneously mixing a specific proportion of crude *Q. acutissima* (acorn jelly powder), *C. sinensis* (dry leaf buds), and *G. thunbergii* (dry aerial part) along with *C. limon* (fruit juice).

*Camellia sinensis* (L.) Kuntze (tea plant) belongs to the family Theaceae (Oliveira et al., 2016). The leaf buds of *C. sinensis* are used to prepare tea, which is the most widely consumed beverage worldwide. It has been used as a traditional medicine to treat urine blockage, wound healing, regulate blood sugar, and improve digestion and mental health. Scientific investigations have demonstrated its multidimensional pharmacological activities with possibilities to be effective in atherosclerosis, hyperlipidemia, various cancers, bowel syndromes, diabetes, liver disease, and obesity (Chopade et al., 2008; Huang et al., 2014; Oliveira et al., 2016). The major portion of chemical constituents in *C. sinensis* is covered with polyphenols. Flavan-3-ols (catechins) are the most abundant active polyphenols present in green tea; hence most of the pharmacological activities offered by green tea are due to the presence of these catechins (Chopade et al., 2008; Huang et al., 2014; Oliveira et al., 2016).

*Quercus acutissima* Carruth., belongs to the family Fagaceae, is a deciduous tree and native to Eastern Asian countries such as Korea, Japan, and Vietnam (Whittemore, 2004). Traditionally, acorns have been consumed to relieve laryngopharyngitis, colitis, labor pain, obesity, and stomatitis. Nutritionally, acorns are composed of starch and other carbohydrates, protein, and fatty acids. Pharmacological studies demonstrated that *Q. acutissima* exhibits antioxidant, anti-obesity, antidementia, anti-diabetic, anti-inflammatory, anti-asthmatic, and protective effects in liver and degenerative diseases (Moon et al., 2012; Vinha et al., 2016).

*Geranium thunbergii* Siebold & Zucc. is a perennial herb, belongs to the family Geraniaceae (Youn and Jun 2013). Traditionally it has been used to treat gastric and intestinal disorders, arthritis, infections, and skin diseases in East Asia. Scientific investigations revealed several pharmacological activities on it such as anti-cancer, anti-mutagenic, anti-inflammatory, anti-oxidant, and anti-obesity activities (Choi et al., 2015; Lee et al., 2020; Wikipedia, 2021). *G. thunbergii* constituted tannins, flavonoids, and lignans primarily. Corilagin, geraniin, and ellagic acid are the major ellagitannins of *G. thunbergii*, which represents about 10% of dried leaves of this herb (Choi et al., 2015; Bastian et al., 2018).

The anti-obesity activity of *C. sinensis* (CS; green tea), *Q. acutissima* (QA; acorn), and *G. thunbergii* (GT) has been individually investigated in previous studies (Sung et al., 2011; Kim et al., 2014; Choi et al., 2016; Muenzner et al., 2016; Hwang et al., 2017). But, the combined effect of these botanical drugs has not been evaluated. The main objective of this study is to develop an herbal formulation (18KHT01) using these natural medicines and evaluate their synergistic anti-obesity activity in obese mice.

## Materials and Methods

### Experimental Materials

The experimental materials, solvents, chemical reagents, cell culture and bioassay reagents, chromatographic supplies, and instrumentals used in this study are noted in the Supplementary Material.

### Preparation and Extraction of 18KHT01

The herb ingredients of 18KHT01 were obtained and identified by Prof. Hyun-Ju Jung, Wonkwang University. The sample specimens are illustrated in Supplementary Figure S1. Lemon was not considered to play a therapeutic role in the formulation. The rationale behind the incorporation of lemon into the 18KHT01 formulation was just as an olfactory supplement. The ratio of each ingredient distributed in crude 18KHT01 is presented in Table 1. The ingredients of 18KHT01 were crushed and ground using mortar and pestle, and homogeneously mixed. The formulation was subjected to extract using 40% ethanol solvent using reflux at 70°C. The schematic diagram of the extraction process is illustrated in Supplementary Figure S2. The dry extract was obtained by freeze-drying. The extraction procedure and parameters were kept constant for the extraction of individual ingredient herbs. The extracts were stored in a refrigerator at 4°C until performing further experiments. The yield values for the individual herb ingredients in 40% ethanol solvent were varied, which altered the final composition of 40% ethanol extract of the 18KHT01 (Supplementary Table S1).

**TABLE 1:** Ingredients and crude composition of 18KHT01.

Ingredients	Part used	Amount distribution (g)
*Quercus acutissima* Carruth. [Fagaceae]	Acorn jelly powder	8.94
*Geranium thunbergii* Siebold & Zucc. [Geraniaceae]	Aerial part	3.74
*Camellia sinensis* (L.) Kuntze [Theaceae]	Leaf buds	5.63
*Citrus limon* (L.) Osbeck [Rutaceae]	Fruit juice (pH: 1.93)	5.59 (5.43 ml)

The 18KHT01 was prepared by homogeneously mixing a specific proportion of raw *Q. acutissima* (QA), *G. thunbergii* (GT), *C. sinensis* (CS) along with fresh lemon juice. The ingredient herbs were crushed using mortar and pestle before mixing.

### Phytochemical Analysis of 18KHT01

#### Preparation of Sample and Standard Solution

Freeze-dried 40% ethanol extract of individual plant ingredients, as well as 18KHT01, were accurately weighed and dissolved in 40% methanol/water to prepare a concentration of 5 mg/ml solution. Stock solution of standard mix containing; caffeine, (-)-epicatechin (EC), Corilagin, (-)-epigallocatechin-3-gallate (EGCG), and (-)-epicatechin-3-gallate (ECG) was prepared in a single vial by dissolving into 40% methanol/water to get concentrations of 500 μg/ml, 250 μg/ml, 250 μg/ml, 1,000 μg/ml, and 500 μg/ml, respectively. Ellagic acid stock solution was separately prepared by dissolving in 100% methanol at concentration of a 300 μg/ml. Each solution was filtered through a 0.2 μm PTFE hydrophilic syringe filter before injection.

### Chromatographic Condition

An Ultra Performance Liquid Chromatography (UPLC) system was used to perform phytochemical analysis. The chromatographic condition and the running method are presented in Supplementary Table S2. Caffeine, corilagin, EC, EGCG, and ECG were analyzed at a UV wavelength of 210 nm whereas ellagic acid was analyzed at 254 nm. A post time of 3 min was set before the next injection. The optimized chromatographic method was subsequently applied for the quality evaluation and simultaneous determination of marker compounds in 18KHT01 and individual ingredients. The marker compounds were quantified by linear regression using standard curves. Each sample was analyzed three times to determine the mean content of the marker compounds.

### Evaluation of *In Vitro* Antioxidant Activity of 18KHT01

Antioxidant activity of 18KHT01 was determined by DPPH and ABTS radical scavenging assays using the method described by Jeong et al. (Jeong et al., 2014). Gallic acid was used as a positive control for standard comparison. The radical scavenging was calculated by using the following equation:

Scavenging Activity (%) = [1-(Abssample/Abscontrol)] × 100

Logarithmic regression curves were plotted by using the percentage of DPPH or ABTS radical scavenging values and treated concentrations and calculated IC_50_ values (quantity of antioxidants necessary to reduce free radicals by 50% concentration). The synergy on 18KHT01 for anti-oxidant activity was calculated in terms of combination index (CI). The CI analysis was carried by using ‘CompuSyn’ computer software, which was based on the Chou-Talalay principle (Chou, 2006).

### Cell Culture and Adipocyte Differentiation

The secondary 3T3-L1 fibroblast cells (ATCC^®^ CL-173TM) of passage number 3–5 were subjected to grow and maintained in DMEM containing 10% NCS with 1% Penicillin-Streptomycin (10,000 units Penicillin; 10,000 µg Streptomycin) in a humidified atmosphere of 5% CO2 at 37°C. MTT cell viability assay and LDH cytotoxicity assay were performed to assess the toxicity of 18KHT01 and its ingredients in 3T3-L1 adipocytes.

Adipocyte differentiation and measurement of lipid accumulation were done according to the method described in the previous study with slight modification (Pandeya et al., 2021). The 3T3-L1 pre-adipocytes were seeded at a density of 5.2 × 10^3^ cells/well of 24 well-plates and 6 × 10^4^ cells/well of 6 well plates in DMEM supplemented with 10% NCS media. The method of cell differentiation and sample treatment is illustrated in Supplementary Figure S3. The 18KHT01 was treated in the dose of 40, 60, and 80 μg/mL. A well-accepted anti-adipogenic compound, EGCG in a dose of 100 μM, was used as a positive control. Some wells were assigned for negative controls (NC), which were treated with 10% FBS/DMEM media without MDI cocktail. At the end of the experiments, the cells were washed with PBS once and fixed in 10% formalin solution for 24 h. The cellular lipid contents were determined by ORO staining. The ORO stained cells were examined using an EVOS XL core light microscope and images were captured. The ORO uptake by lipid content into cells was extracted with 100% isopropanol solvent and the absorbance was measured at 520 nm using a micro-plate reader. The amount of lipid accumulation into adipocytes was calculated and expressed as a percentage of the control.

To measure the synergistic anti-adipogenic activity of 18KHT01, the 3T3-L1 adipocytes were treated by two methods illustrated in Supplementary Figure S4. In the first method, 18KHT01 along with its all ingredients; CS, QA, GT, and lemon were treated at an equal concentration of 80 μg/ml, and activities were compared. In the second method, an 80 μg/ml of 18KHT01 and equivalent amounts of ingredients present on the 80 μg/ml concentration of 18KHT01 as the composition of the 18KHT01 ethanol extract (Supplementary Table S1), were separately treated and cumulative inhibition of lipid production by the ingredients was compared with the activity by 18KHT01 alone. The anti-adipogenic activity of the formulation and its ingredients were evaluated by the ORO staining method.

### Ribonucleic Acid Extraction and Real-Time Polymerase Chain Reaction Analysis

The total RNA extraction, cDNA synthesis, and real-time PCR analysis were performed on 18KHT01 treated 3T3-L1 adipocytes according to the previously described methods (Pandeya et al., 2019). The 3T3-L1 adipocytes were washed with ice-cold PBS and harvested in an RNA extraction reagent. The total RNA from the 3T3-L1 adipocytes were separately extracted. A total of 1 μg mRNA was reverse-transcribed into cDNA using a High-capacity RNA-to-cDNA kit. The gene expression levels were analyzed by quantitative real-time PCR. The primer sequences used for RT-PCR are presented in the Supplementary Table S4. The mouse β-actin was used as a reference gene. Relative mRNA expression levels were calculated using the Livak ΔΔCt method (Livak and Schmittgen, 2001).

### Experimental Animals and Diets

Six-week-old male C57BL/6J mice (20–22 g) were supplied by Central Lab. Animal Inc., Seoul, Korea. The animals were housed and maintained in a standard laboratory condition (room temperature: 24 ± 1°C, relative humidity: 50–60%, light cycle: 7:00–19:00). The animal study was approved by the Animal Experiment Ethical Committee of the Wonkwang University (Approval number: WKU19-76). The mice were acclimatized to their environment for 1 week before the commencement of the experiments.

Mice were fed with either a standard chow diet (Standard diet: 5L79 Orient Bio Inc., Seongnam, Korea) consisting of 13.67% fat; 65.30% carbohydrate; and 20.1% protein or a high-fat diet (Rodent Diet D12492, Research Diets, New Brunswick, NJ, United States) consisting of 60% fat; 20% carbohydrate; and 20% protein. The compositions of experimental diets used in this study are presented in Supplementary Table S6. Mice were allowed free access to feed and water.

### Animal Studies and Mice Grouping

Two successive animal studies were carried to investigate the anti-obesity activity of 18KHT01 and to determine the synergistic effect of the formulation. To study the anti-obesity activity, diet-induced obesity (DIO) was developed in mice before drug treatment.

#### Development of Diet-Induced Obesity Mice Model and Drug Treatments

To determine the anti-obesity activity of 18KHT01, mice were randomly divided into five groups (*n* = 6). A normal group was fed with the standard chow diet whereas the remaining four groups were fed with a high-fat diet (HFD) for 6 weeks without drug treatments. In the 6 weeks (obesity induction period), the mice feeding a high-fat diet were considered to be obese (the average body weight was more than 42% higher than the normal mice body weight), indicating a successful development of the DIO model in mice. Thereafter, the diet-induced obese mice were treated with various samples or blank for another 11 weeks (drug treatment period) with a continuation of high-fat diets. Normal and high-fat diet control (HFD) groups were treated with vehicle (0.2% CMC/PBS) orally. Standard orlistat (PHR1445-1G, Sigma-aldrich, United States) at a dose of 10 mg/kg, twice daily was given as a positive control to a group. As the 18KHT01 formulation was found to be safe below 500 mg/kg/day on sub-acute treatment in mice from the preliminary toxicity study (data not shown), the remaining two DIO groups were treated with 18KHT01 in the dose of 75 mg/kg and 150 mg/kg, twice daily by oral gavage. The doses of the formulation were increased by 5% in every 3 weeks but were not exceed 15%. Food intake and body weights were recorded every week. The fasting blood glucose was recorded from the tail blood on the first day of the experiment before switching normal diet to a high-fat diet (Week 0), on the first day of drug treatment (Week 6), and in every 4 weeks interval thereafter. The mice were kept fasting for 17–18 h before measuring blood glucose. At the end of the experiment, mice were sacrificed in fasting condition and blood samples were collected from the retro-orbital puncture and all mice were euthanized by cervical dislocation.

#### Assessment of Synergistic Anti-Obesity Activity of 18KHT01 in Mice

To determine the synergistic activity of 18KHT01, the animals were randomly divided into seven groups each containing five mice and the experimental diets were provided. A normal group was fed with the standard chow diet. The remaining six groups were fed with HFD. Drug treatments were initiated from the same day of switching mice to an HFD feed. One of the groups from HFD fed mice was separated as a control. The normal and HFD control groups were orally treated with vehicle. Orlistat was treated to a group as a positive control. The remaining four HFD fed groups were separately treated with 40% ethanol extracts of 18KHT01, *C. sinensis* (CS), *Q. acutissima* (QA), and *G. thunbergii* (GT) in an equal dose of 150 mg/kg, twice daily through oral gavage. The doses of each experimental sample were increased by 5% in every 3 weeks but were not exceed 15%. Drug treatments were carried for 9 weeks. The body weight and food intake were recorded every week. Animals were monitored twice daily at the time of drug treatment for any pathological changes. The fasting blood glucose was measured in every 3 weeks. Before sacrifice, blood samples were collected into separate vials for hematological analysis and serum separation. As the lemon did not reveal activity in antioxidant and anti-adipogenic assessments, it was not considered to play a therapeutic role in the formulation. Thus, it was excluded from the animal experiment.

### Measurement of Serum Insulin and Determination of HOMA-IR

The serum insulin was determined by using Mouse Insulin ELISA Kit (Shibayagi Co., Japan) according to the manufacturer’s protocol. The HOMA-IR was calculated using fasting blood glucose value measured at the sacrifice day and the serum insulin concentration according to the equation as follows (Muniyappa et al., 2008).

HOMA-IR = [fasting glucose (mmol/l) × fasting insulin (μU/mL)]/22.5

### Serum Analysis

The sera were separated by centrifugation of collected blood samples (14,000 rpm for 20 min at 4°C) and then stored at −70°C for further analysis. The level of TC, LDL/VLDL, HDL, TG, creatinine, total bilirubin, and ALT and AST activities in serum were analyzed by using respective colorimetric assay kits (BioVision, Milpitas, United States) according to the manufacturer’s procedure.

Atherogenic index (AI) was assessed to analyze the development of dyslipidemia in the mice model. The AI and the percentage of protection offered by the study drugs were calculated as follows (Munshi et al., 2014):

Atherogenic index (AI) = (Total cholesterol–HDL)/HDL

Protection (%) = (AI of HFD control – AI of treatment group)/AI of HFD control × 100

### Histological Analysis

The epididymal white adipose tissue, liver, kidney, and spleen were isolated, weighed, and immediately fixed into 10% formalin with PBS for histological analysis. Hematoxylin and eosin (H&E) staining was done for histological analysis according to the method described in the previous report (Pandeya et al., 2021). Micrographs were analyzed using an EVOS XL core light microscope.

### Statistical Analysis

Statistical significance among the experimental groups was analyzed with GraphPad Prism 7 software using a one-way analysis of variance (ANOVA) followed by Dunnett’s multiple range tests. In the synergy evaluation animal study, the *G. thunbergii* (GT) treated group was excluded from statistical analysis due to remaining only two mice in the group after the death of toxicity. All data are presented as the mean ± standard deviation (SD). *p*-Values less than 0.05 were considered statistically significant.

## Results

### Phytochemical Analysis of 18KHT01

**FIGURE 1:**
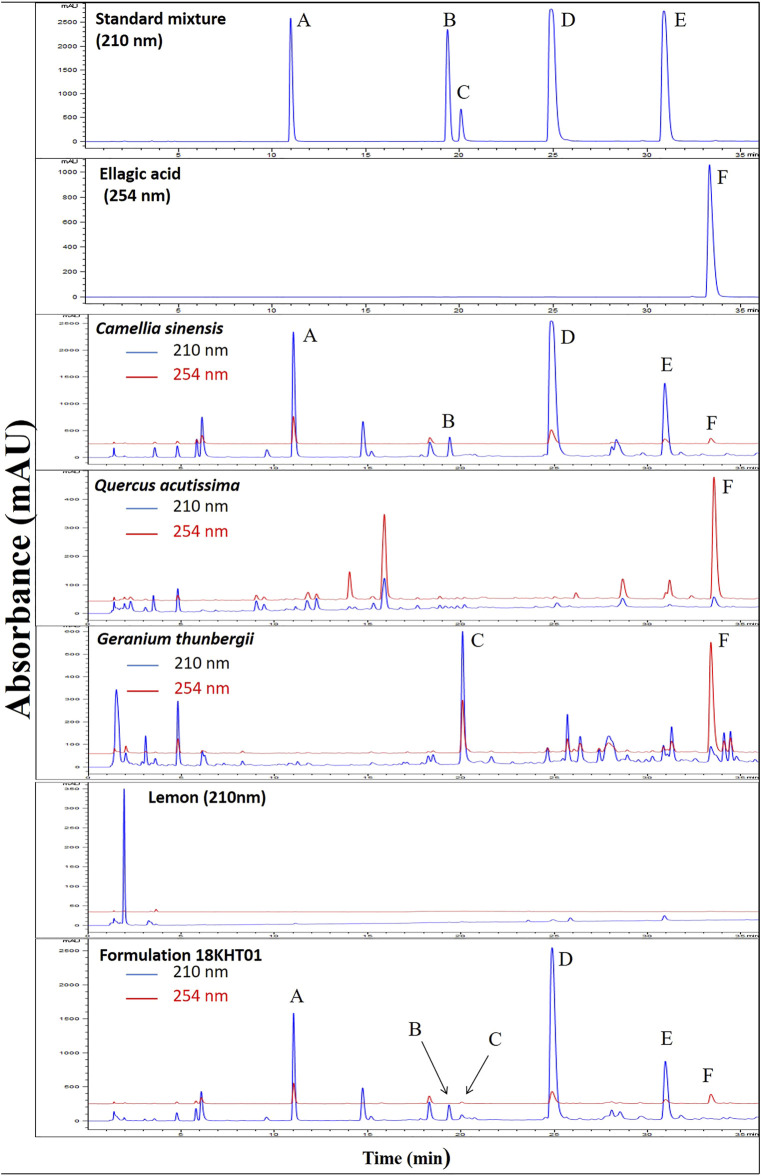
UPLC chromatogram of standards, individual botanical drug ingredients, and 18KHT01 at 210 nm (blue) and 254 nm (red). The marker compounds are **(A)** caffeine **(B)** epicatechin **(C)** corilagin **(D)** EGCG **(E)** ECG; and **(F)** ellagic acid.

The formulation 18KHT01, its plant ingredients, and standard compounds were subjected to run using the chromatographic method presented in Supplementary Table S2. The typical UPLC chromatograms of the standard mixture, individual botanical drug ingredients, and 18KHT01 are shown in Figure 1. Caffeine, corilagin, EC, EGCG, and ECG were detected at a wavelength of 210 nm whereas ellagic acid was detected at 254 nm. The retention time for caffeine, EC, corilagin, EGCG, ECG, and ellagic acid in 18KHT01 was observed to be 10.69 ± 0.02, 19.19 ± 0.03, 19.92 ± 0.02, 24.93 ± 0.02, 31.09 ± 0.02, and 33.28 ± 0.02 min, respectively. Similarly, the content of these marker compounds in 18KHT01 were found to be 54.89 ± 0.43, 3.32 ± 0.04, 6.07 ± 0.06, 155.70 ± 1.35, 24.50 ± 0.21, and 12.26 ± 0.086 mg/g, respectively.

### 18KHT01 Showed Synergistic Antioxidant Activity *In Vitro*

The DPPH and ABTS antioxidant activities of 18KHT01 and its ingredients are expressed in terms of IC_50_ and presented in Table 2. Results showed that 18KHT01 had better antioxidant activity than that of *Q. acutissima*, *G. thunbergii*, and lemon juice, and was comparable with that of *C. sinensis*, which indicated that a small amount of the ingredients showed similar or better antioxidant activity if they were treated in a combination as 18KHT01.

**TABLE 2:** IC_50_ values for the antioxidant activities of 18KHT01 and its ingredients.

Samples	DPPH-IC_50_ (µg/ml)	ABTS-IC_50_ (µg/ml)
18KHT01	2.00	1.96
*Q. acutissima*	10.17	10.37
*G. thunbergii*	4.30	3.92
*C. sinensis*	1.84	1.63
*C. limon* Juice	NA	NA
Gallic acid	0.80	0.72

The IC_50_ values were determined by interpolation from the logarithmic regression of concentration of samples against percentage inhibition. NA , not active.

The synergistic interactions among ingredients in the 18KHT01 were analyzed and presented in terms of combination index (CI) value. The CI values and their significance at different concentrations for DPPH and ABTS antioxidant activities are presented in Supplementary Table S3. It was observed that the CI values were less than 1 at most of the higher concentrations, in-fact, showed the lowest value at a 15.625 μg/ml on ABTS assay. The overall results suggested that a strong synergistic effect of 18KHT01 for antioxidant activity.

### 18KHT01 Showed Synergistic Anti-adipogenic Activity in 3T3-L1 Adipocytes

**FIGURE 2:**
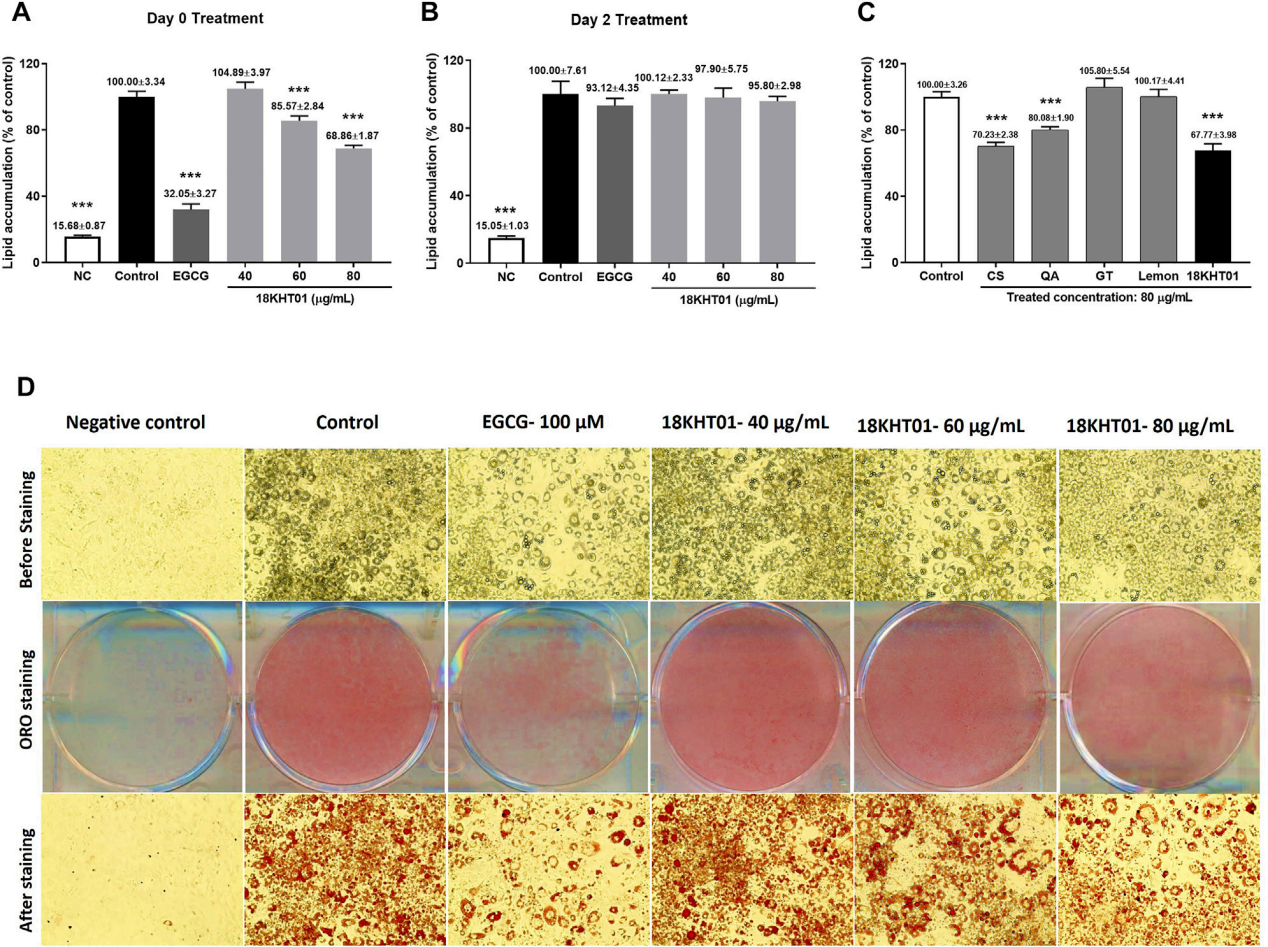
Effect of 18KHT01 on differentiation of 3T3-L1 adipocytes. The 3T3-L1 pre-adipocytes were seeded in separate plates. The cells of one plate were treated with samples at **(A)** Day 0 by mixing with MDI cocktail and another at **(B)** Day 2 with insulin media. **(C)** The 18KHT01 and its ingredients at an equal concentration of 80 μg/ml were separately treated to evaluate synergistic effect. The amounts of lipid accumulations were quantified by Oil red O (ORO) assay. **(D)** The lipid accumulation in the adipocytes was visualized before and after ORO staining using a light microscope and images were captured at ×20 magnification. Statistical significance was calculated using one-way ANOVA followed by Dunnett’s multiple comparisons test. The data shown are represented as mean ± SD of four separate experiments with significance ***p < 0.001 vs control. NC = Negative control; Control = MDI control (Blank treated); EGCG = (-)-Epigallocatechin-3-gallate (100 µM); CS = *Camellia sinensis*; QA = *Quercus acutissima*; GT = *Geranium thunbergii*; Lemon = *Citrus limon*.

The 18KHT01, and its ingredients were found to be safe below the concentration of 80 μg/ml to be treated in 3T3-L1 adipocytes (Supplementary Figure S5 and Supplementary Table S5). The adipocytes were separately treated at Day 0 and Day 2 of the differentiation period to identify the effect of 18KHT01 on different stages of adipogenesis. The amounts of the lipid accumulation in the adipocytes were expressed as a percentage of the control (Figures 2A,B). As shown in Figure 2A, 18KHT01 was found to be effective in the inhibition of adipocyte differentiation only if treated at Day 0 with adipogenesis induction (MDI) media. The results indicated that 18KHT01 had a preventive effect on adipogenesis. The adipocytes were visualized before and after the ORO staining and photographed using a light microscope. As shown in Figure 2D, lipid accumulation was largely suppressed by EGCG-100 µM and 18KHT01–80 μg/ml compared to the control.

Evaluation of synergistic anti-adipogenic activity revealed that the ingredients of 18KHT01 showed the best synergy when treated in the form of combination rather than treating separately. When treating in an equal concentration of 80 μg/ml of 18KHT01 and its all ingredients, the 18KHT01 exhibited the lowest degree of lipid accumulation (Figure 2C). The 18KHT01 extract was composed of small proportions of these ingredients. Therefore, the results suggested that a small amount of the ingredients showed higher activity when they were combined as the formulation. In addition, a cumulative lipid inhibition by all the ingredients of 18KHT01 was significantly lowered than that by separately treated 18KHT01 formulation (Table 3).

**TABLE 3:** Synergistic anti-adipogenic effect of 18KHT01 on 3T3-L1 adipocytes.

18KHT01 and ingredients	Amount of ingredients in 80 μg/ml of 18KHT01	Lipid accumulation (%)	Lipid inhibition (%)
Control	-	100.00 ± 4.28	0.00 ± 4.28
CS	43.90 μg/ml	92.28 ± 4.60	7.72 ± 4.60
QA	9.43 μg/ml	98.60 ± 4.07	1.40 ± 4.07
GT	17.02 μg/ml	110.77 ± 6.51	−10.77 ± 6.51
Lemon	9.64 μg/ml	99.83 ± 5.64	0.17 ± 5.64
18KHT01	80 μg/ml	70.91 ± 2.89	29.09 ± 2.89
Cumulative inhibition of lipid production by the ingredients of 18KHT01 = −1.48			

The synergistic effect of 18KHT01 due to its ingredients on relative lipid accumulation on 3T3-L1 adipocytes was determined by ORO staining. The lipid accumulations (expressed as the percentage of control) are presented as means ± SD, of four separate experiments. Control = MDI, control (Blank treated); CS, *camellia sinensis*; QA, *quercus acutissima*; GT, *geranium thunbergii*; Lemon = *Citrus limon*.

### 18KHT01 Downregulated Adipogenic Genes in 3T3-L1 Adipocytes

**FIGURE 3:**
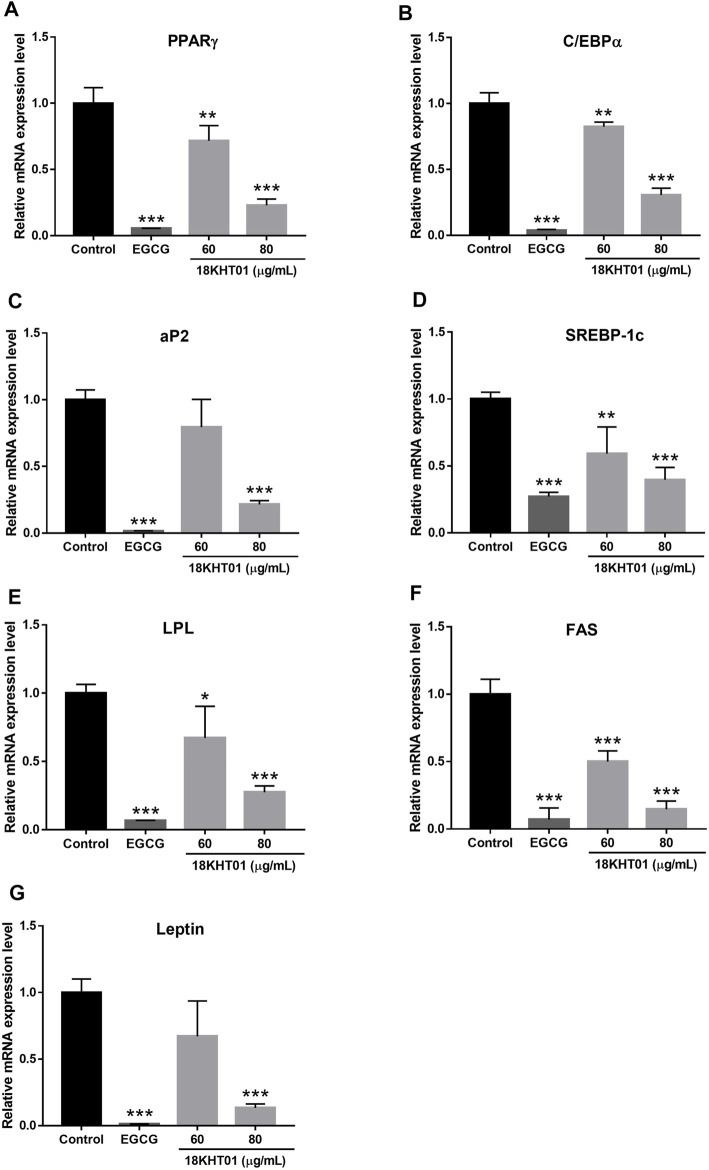
Effect of 18KHT01 on mRNA expression of adipogenic factors in 3T3-L1 adipocytes. The 3T3-L1 pre-adipocytes were treated with or without 18KHT01 (60 and 80 μg/ml) and EGCG (100 µM) with MDI media. A total of RNAs were extracted on the 7th day of the treatment. Gene expression levels of key adipogenic markers were determined. **(A)** PPARγ **(B)** C/EBPα **(C)** aP2 **(D)** SREBP-1c **(E)** LPL **(F)** FAS **(G)** Leptin. Each gene expression level was normalized to β-actin. Statistical significance was calculated using one-way ANOVA followed by Dunnett’s multiple comparisons test. The data shown are represented as mean ± SD (*n* = 3) with significance *p < 0.05, **p < 0.01, ***p < 0.001 vs control. Control = MDI control; EGCG = (-)-Epigallocatechin-3-gallate.

Differentiation of adipocytes required wide-ranging networking and sequential activation of numerous adipogenic factors such as PPARγ, C/EBPα, aP2, SREBP-1c, LPL, and FAS (Moseti et al., 2016). Thus, the effects of 18KHT01 on the mRNA expression of these adipogenic markers were analyzed by RT-PCR. The mRNA expression levels of the aforementioned adipogenic markers were significantly down-regulated by 18KHT01 in a concentration-dependent manner (Figure 3). In addition, 18KHT01 also down-regulated the expression of an adipokine, leptin, in 3T3-L1 adipocytes (Figure 3G). Thus, the result suggested that 18KHT01 inhibited adipocyte differentiation through the downregulation of these adipogenic factors.

### 18KHT01 Regulated Body Weight in Obese Mice

**FIGURE 4:**
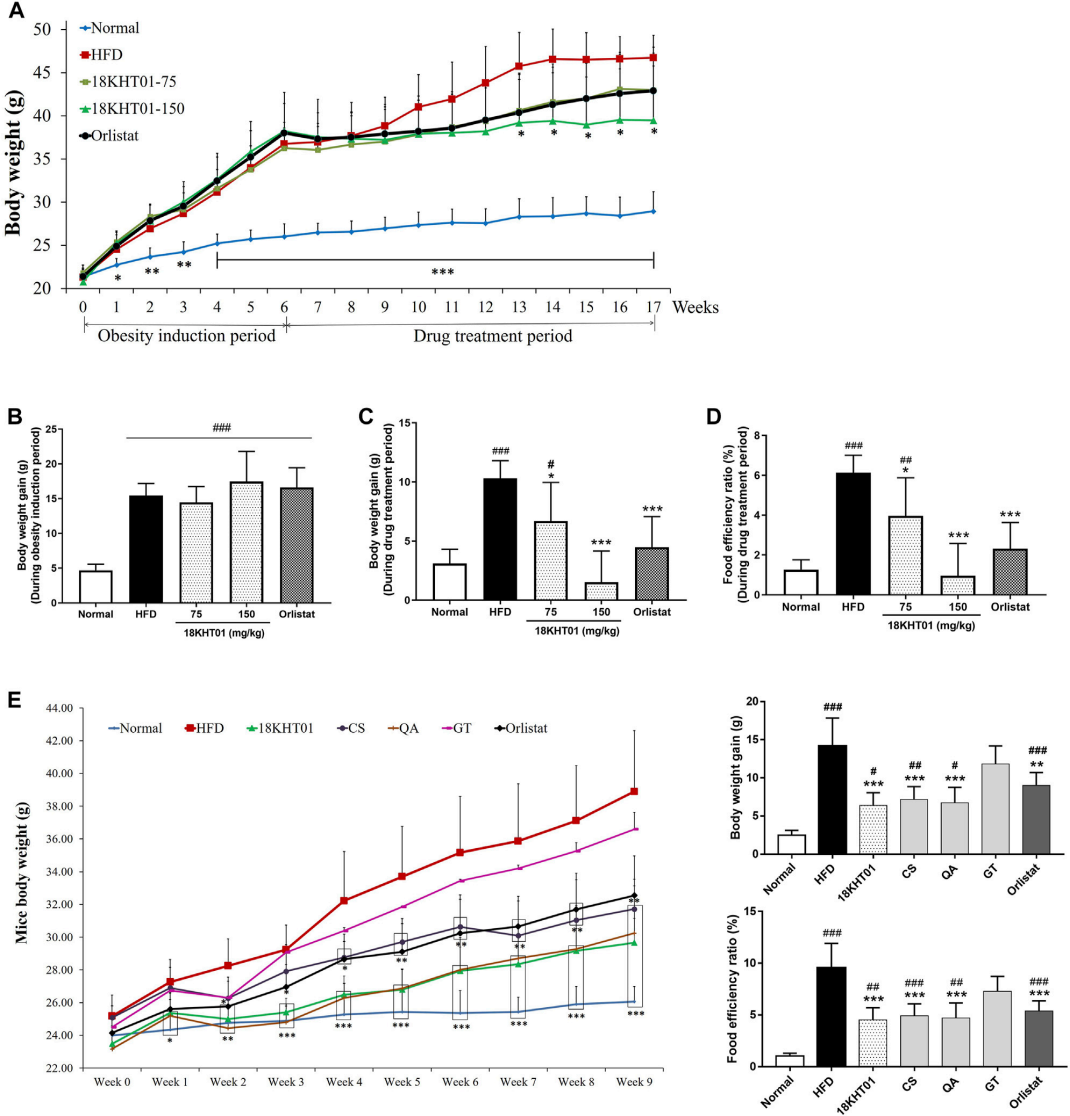
Effect of 18KHT01 on body weights of obese C57BL/6J mice. Mice body weights were measured every week over the experimental period. **(A)** Body weight pattern of mice during the obesity induction period and drug treatment period. **(B)** Body weight gain during 6 weeks of the obesity induction period and **(C)** weight gain during 11 weeks of the drug treatment period. **(D)** Food efficiency ratio (FER) during 11 weeks of drug treatment period. The food efficiency ratio (FER) was calculated as follows: FER% = gained body weight (g) × 100/food intake (g). **(E)** The effects of 18KHT01 and its ingredients on body weight pattern, weight gain, and FER during 9 weeks treatment in synergy evaluation study. Statistical significance was calculated using one-way ANOVA followed by Dunnett’s multiple comparisons test. Data are expressed as mean ± standard deviation (*n* = 6 or 5) with significance #*p* < 0.05, ##*p* < 0.01, ###*p* < 0.001 vs Normal; *p < 0.05, **p < 0.01, ***p < 0.001 vs HFD control. Normal = Standard chow diet; HFD = high-fat diet control; Orlistat = orlistat (10 mg/kg); CS = *Camellia sinensis* (150 mg/kg); QA = *Quercus acutissima* (150 mg/kg); GT = *Geranium thunbergii* (150 mg/kg).

In this study, the DIO mice model has been developed in C57BL/6J mice before evaluation of the anti-obesity activity of 18KHT01. Body weights were measured from starting of the experiment in a weekly basis. Mice body weight pattern in obesity induction period as well as drug treatment period is shown in Figure 4A. During the 6 weeks of the obesity induction period, the body weight gain was significantly increased in four high-fat diet-fed groups in comparison to the standard diet-fed normal group (Figure 4B). The body weight gain in the HFD control group remained significantly higher compared to the normal group during the drug treatment period as well. However, the 18KHT01 and orlistat treated groups significantly reduced the weight gain in comparison to the HFD group (Figure 4C). The weight gain in 18KHT01 (150 mg/kg) and orlistat groups were not statistically differed to the normal. It showed that the 18KHT01 had potent body weight-reducing activity in obese mice. Compared to the HFD control group, the body weight was found to be significantly reduced in 150 mg/kg of 18KHT01 treated group after 7th weeks of treatment (Figure 4A). High energy consumption is a critical factor in the development of obesity. The food intakes were evaluated every week to measure the amount of energy consumption by mice. The food intake patterns among high-fat diet-fed groups were found to be similar during the obesity induction period as well as the drug treatment period (Supplementary Figures S6A,B). The food efficiency ratio (FER) over the obesity induction period was similar in all high-fat diet-fed groups and was significantly higher in comparison to the normal group (Supplementary Figure S6C). Assessment of FER during the drug treatment period revealed that the HFD control group remained significantly higher compared to the normal. However, the 18KHT01 and orlistat significantly reduced FER compared to the HFD control (Figure 4D). In comparison to the normal group, the 150 mg/kg of 18KHT01 and orlistat treated groups showed no significant difference in FER during the drug treatment period. This result indicated the weight gaining pattern in the 18KHT01 treated group was similar to a normal group even providing a high-fat diet.

Evaluation of synergistic anti-obesity activity revealed the 18KHT01 as a potent therapy among all the treated samples in reduction of mice body weight. In addition, the extent of controlling of weight gain and FER were observed to be most effective in 18KHT01 treatment among all the treatment groups (Figure 4E).

### 18KHT01 ameliorated Fasting Blood Glucose Level and Insulin Resistance in Obese Mice

**FIGURE 5:**
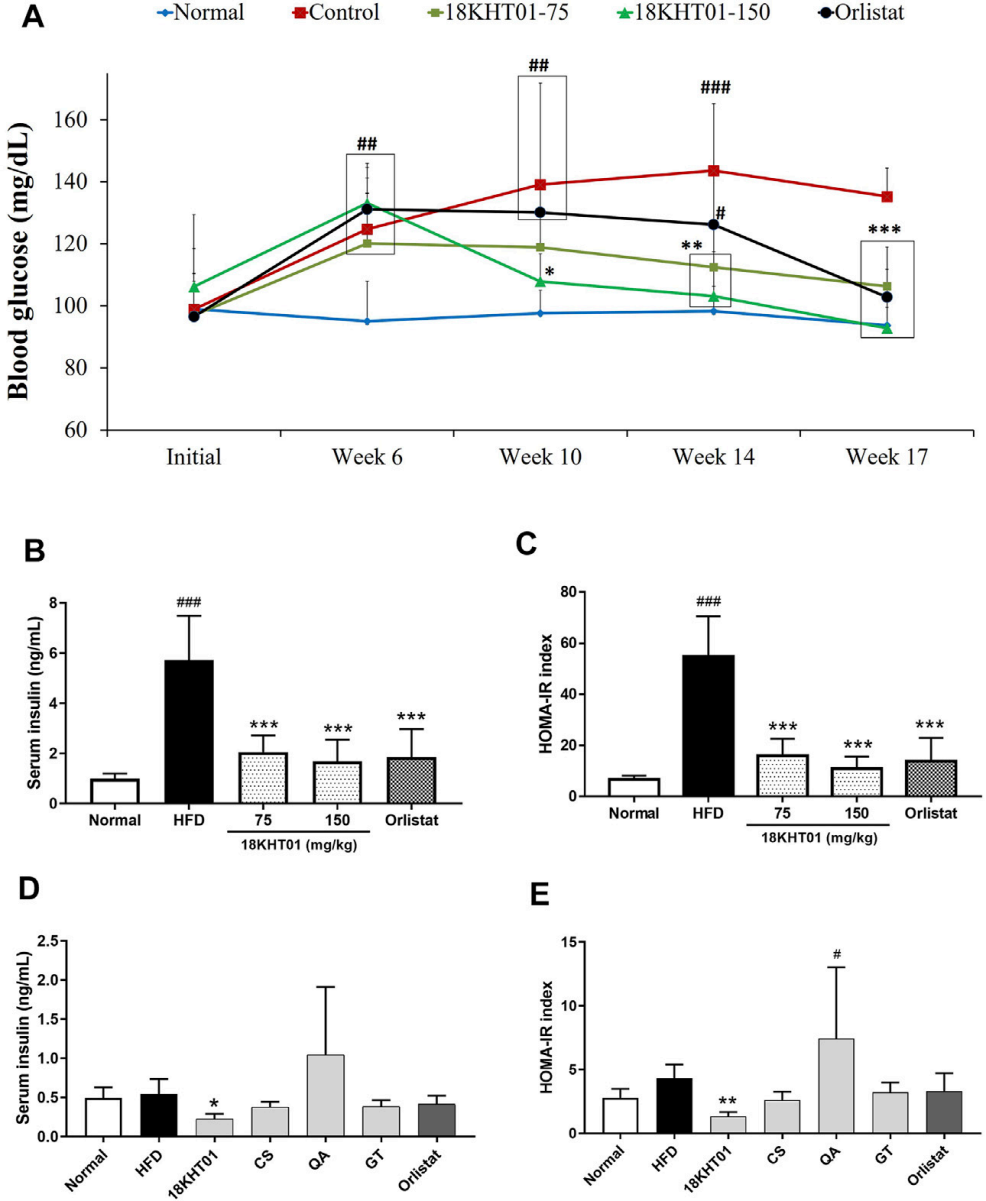
Effect of 18KHT01 on fasting blood glucose and insulin level. **(A)** Fasting blood glucose level over the experimental period. **(B)** Serum insulin level. **(C)** HOMA-IR index. HOMA-IR index was calculated as follows: HOMA-IR = [fasting insulin (μU/mL) × fasting glucose (mmol/l)]/22.5. In the synergy evaluation animal study **(D)** serum insulin concentration, and **(E)** HOMA-IR index of each experimental group was also determined. Statistical significance was calculated using one-way ANOVA followed by Dunnett’s multiple comparisons test. Values are expressed as mean ± standard deviation (n = 6) with significance #*p* < 0.05, ##*p* < 0.01, ###*p* < 0.001 vs Normal; *p < 0.05, **p < 0.01, ***p < 0.001 vs HFD control. Normal = Standard chow diet; HFD/Control = high-fat diet control; Orlistat = orlistat (10 mg/kg); CS = *Camellia sinensis* (150 mg/kg); QA = *Quercus acutissima* (150 mg/kg); GT = *Geranium thunbergii* (150 mg/kg).

Blood glucose was measured in a 17–18 h fasting state to determine the effect of 18KHT01 on obesity-induced hyperglycemia. As shown in Figure 5A, the blood glucose level during the 6 weeks of obesity induction period was significantly elevated in four high-fat diet-fed groups compared to the normal group indicating the development of obesity-induced hyperglycemia. The fasting blood glucose in HFD control remained significantly higher over the drug treatment periods compared to the normal. However, 150 mg/kg of 18KHT01 treated group started to show a significant reduction of blood glucose from the 4th week of the treatment (from Week 10) in comparison to the HFD control. At the end of the experiment, mice treated with 150 mg/kg of 18KHT01 showed similar fasting blood glucose levels as the normal group. This result revealed that the 18KHT01 showed potent blood glucose-reducing activity. Hence, 18KHT01 might have a beneficial effect in the management of diabetes as well. The high fasting glucose level might associate with insulin resistance. Over consumption of a fatty diet induces insulin resistance in mice (Hariri and Thibault, 2010). Serum insulin was determined at the end of the experiment. As shown in Figure 5B, the drastically elevated insulin level after 17-weeks consumption of HFD was significantly reduced in 18KHT01 and orlistat-treated groups. Calculation of the HOMA-IR index revealed that significantly high extent in the HFD control group compared to the normal indicating development of insulin resistance. However, the 18KHT01 and orlistat groups showed a significantly low value of the HOMA-IR index in comparison to the HFD control group. The results suggested that an 11 weeks treatment of 18KHT01 ameliorated obesity-induced insulin resistance.

In the synergy evaluation *in vivo* experiment, the fasting blood glucose was measured in every 3-week interval. The 18KHT01 was found to be most effective in reducing fasting blood glucose among all the treated samples. Even providing the HFD, blood glucose levels remains nearly constant over the experimental period in the 18KHT01 treated group, which was comparable with the standard diet-fed normal group (Table 4). Similarly, serum insulin concentration and HOMA-IR index were observed to be decreased significantly only in the 18KHT01 treated group (Figures 5D,E). These observations suggested that a strong synergistic activity of 18KHT01 in ameliorating high-fat diet-induced insulin resistance.

**TABLE 4:** Effect of 18KHT01 and its ingredients on fasting blood glucose level.

Groups	Week 0	Week 3	Week 6	Week 9
Normal	76.6 ± 8.6	60.0 ± 11.9	73.2 ± 3.8	80.6 ± 12.0
HFD Control	83.0 ± 14.0	102.4 ± 20.2^##^	113.6 ± 17.6^###^	115.4 ± 14.1^###^
18KHT01	67.6 ± 7.7	89.2 ± 11.3^#^	90.6 ± 9.3*	79.2 ± 5.0***
*C. sinensis* (CS)	75.0 ± 10.5	98.2 ± 9.5^##^	96.2 ± 13.0^#^	89.0 ± 11.8**
*Q. acutissima* (QA)	79.4 ± 22.3	106.8 ± 28.8^###^	109.0 ± 11.3^##^	104.0 ± 11.7^#^
*G. thunbergii* (GT)	84.6 ± 21.5	124.0 ± 9.9	125.0 ± 17.0	117.0 ± 4.2
Orlistat	75.6 ± 19.0	87.4 ± 8.2^#^	108.6 ± 13.8^###^	104.0 ± 9.8^#^

Blood glucose was measured in every 3 weeks interval in a fasting condition during *in vivo* synergy evaluation study. The results are expressed in mg/dL. Statistical significance was calculated using one-way ANOVA, followed by Dunnett’s multiple comparisons test. Values are presented as the mean ± SD (*n* = 5) with significance #*p* < 0.05, ##*p* < 0.01, ###*p* < 0.001 vs Normal; **p < 0.01, ***p < 0.001 vs HFD, group. Normal = standard diet; HFD, high-fat diet control; CS, *Camellia sinensis* (150 mg/kg); QA, *Quercus acutissima* (150 mg/kg); GT, *Geranium thunbergii* (150 mg/kg); Orlistat = orlistat (10 mg/kg).

### 18KHT01 Regulated Serum Lipid Profile in Obese Mice

**FIGURE 6:**
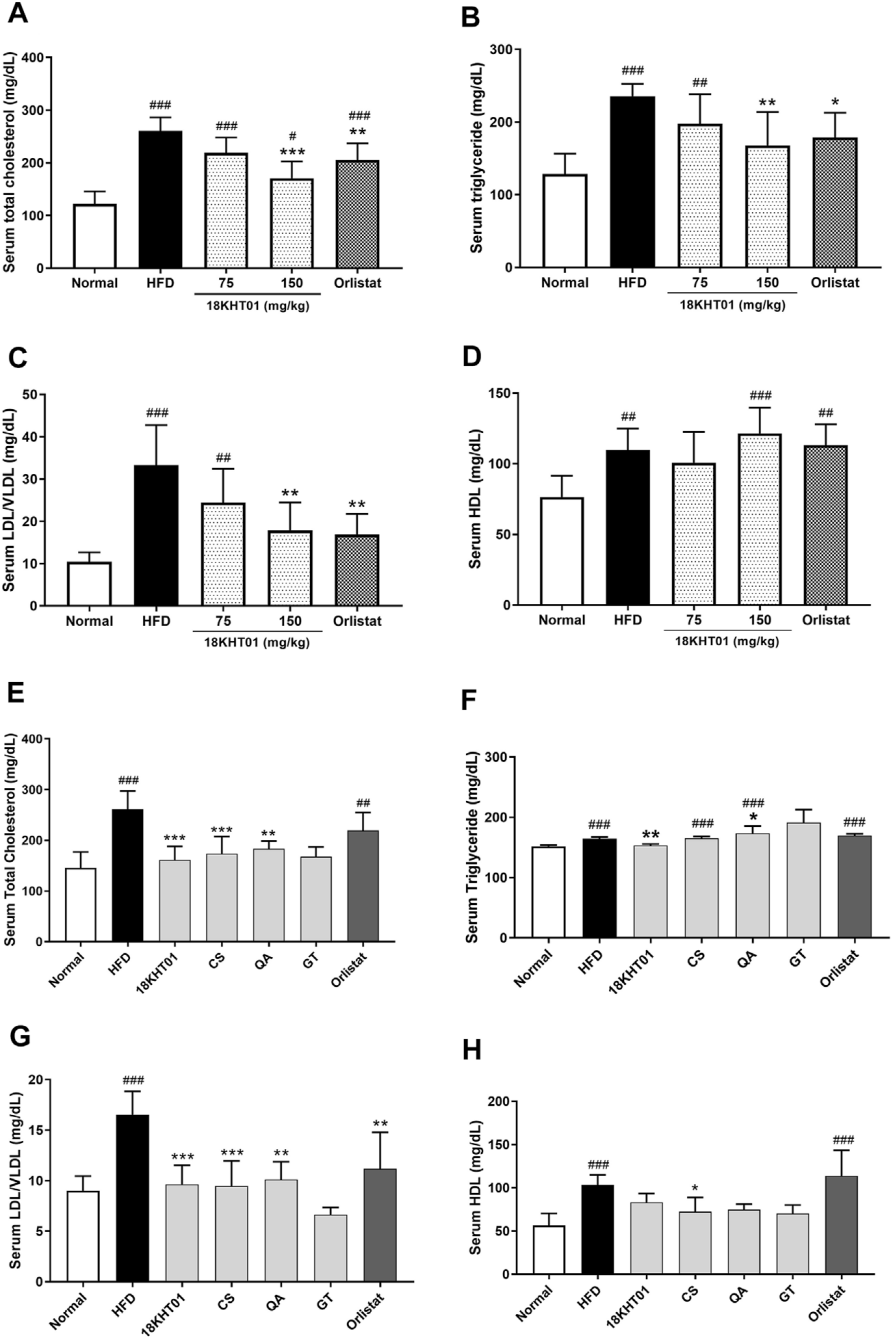
Effect of 18KHT01 on serum lipid profile. Serums lipid profile assay was performed to measure total cholesterol (TC), triglyceride (TG), low/very low-density lipoprotein (LDL/VLDL) and high-density lipoprotein (HDL). In the anti-obesity study, **(A)** serum cholesterol; **(B)** serum triglyceride; **(C)** serum LDL/VLDL; and **(D)** serum HDL were analyzed in DIO mice. Similarly, **(E)** serum TC, **(F)** TG, **(G)** LDL/VLDL, and **(H)** HDL were analyzed in the synergy evaluation animal study. Statistical significance was calculated using one-way ANOVA followed by Dunnett’s multiple comparisons test. Values are expressed as mean ± standard deviation (*n* = 6) with significance #*p* < 0.05, ##*p* < 0.01, ###*p* < 0.001 vs Normal; *p < 0.05, **p < 0.01, ***p < 0.001 vs HFD control. Normal = Standard chow diet; HFD = high-fat diet control; Orlistat = orlistat (10 mg/kg).

Serum lipid profiles were determined by evaluating TC, LDL/VLDL, HDL, and TG. Consumption of a high-fat diet for 17 weeks significantly increased the serum level of the aforementioned lipids indicating the development of hyperlipidemia (Figures 6A–D). Treatment of 18KHT01 and orlistat for 11 weeks significantly lowered the serum TC, TG, and LDL level in obese mice (Figures 6A–C). The results suggested that the 18KHT01 might have a beneficial effect in the management of dyslipidemia. However, the serum level of HDL was not significantly altered with the treatment of 18KHT01 and orlistat compared to the HFD control (Figure 6D). Atherogenic index (AI) indicates the risk of oxidative damage in the heart, coronaries, aorta, liver, and kidney by fatty infiltration or lipid deposition (Munshi et al., 2014). The AI and percentage of protection offered by the drugs are presented in Supplementary Table S7. The 18KHT01 and orlistat treated groups showed significantly lower AI and thus exhibited a high level of protection percentage indicating a protective effect against oxidative stress in visceral organs.

In the synergy evaluation study, the serum TC and LDL/VLDL elevated by HFD feeding were significantly reduced with treatment of 18KHT01 and its ingredients (Figures 6E,G). However, a TG level, which was observed to be elevated in all of the ingredient treated groups, was significantly reduced only in the 18KHT01 treated group and seen in the base level as the normal (Figure 6F). This is an important evidence of showing synergistic activity by 18KHT01 in the reduction of high-fat diet-induced hyperlipidemia.

### 18KHT01 Inhibited Lipid Accumulation Into Liver and White Adipose Tissue

**FIGURE 7:**
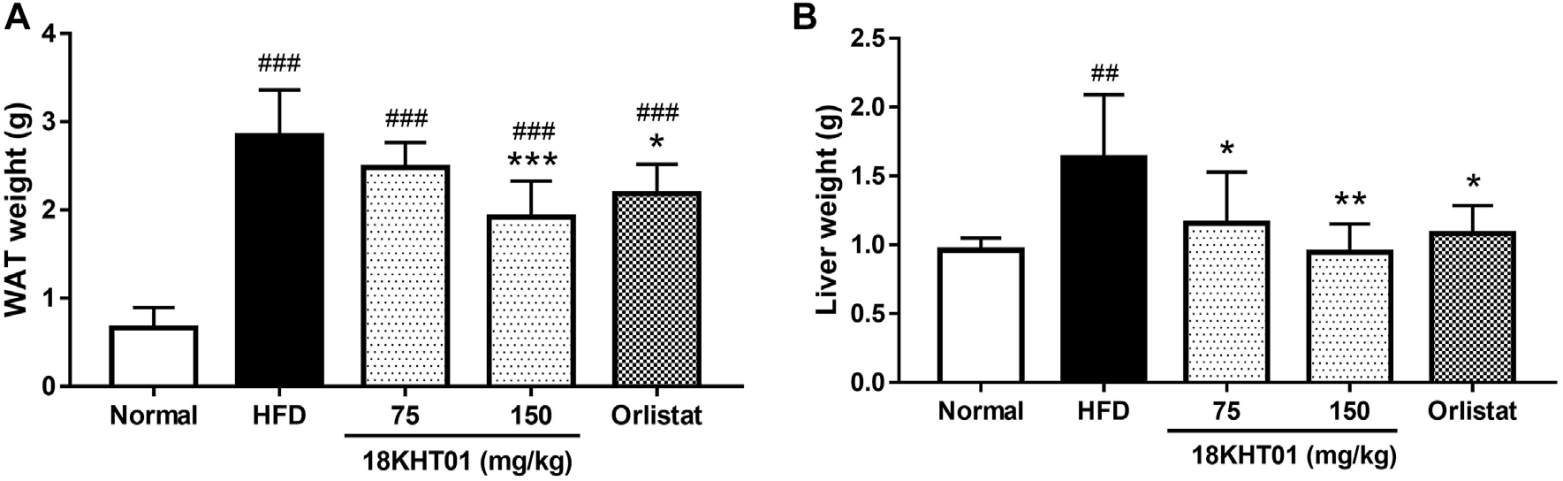
Effect of 18KHT01 on white adipose tissue (WAT) and liver weight. **(A)** Weight of WAT and **(B)** weight of the liver form different group mice. Statistical significance was calculated using one-way ANOVA followed by Dunnett’s multiple comparisons test. Values are expressed as mean ± standard deviation (*n* = 6) with significance ##*p* < 0.01, ###*p* < 0.001 vs Normal; *p < 0.05, **p < 0.01, ***p < 0.001 vs HFD control. Normal = Standard chow diet; HFD = high-fat diet control; Orlistat = orlistat (10 mg/kg).

**FIGURE 8:**
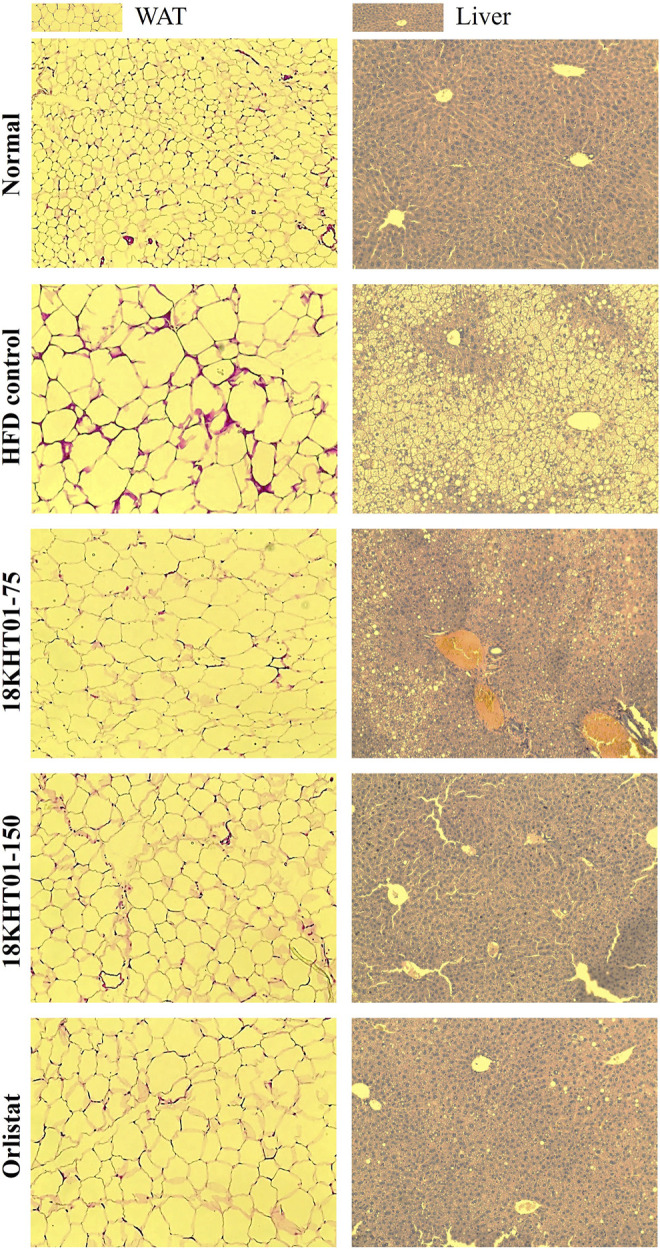
Effect of 18KHT01 on white adipose tissue (WAT) and liver histology. Hematoxylin and eosin (H&E) staining was done to perform histological analysis. Pictures were captured at ×20 magnification under a light microscope. Size of adipocytes and crown-like structure (red eosin stain) were evaluated in WAT. Accumulation of lipid droplets (yellow droplets) and macrophage infiltration are evaluated in the liver. Lipid droplets are more noticeable in HFD control liver.

The liver and epididymal WAT were considered to be involved in energy metabolism. The consumption of a high-fat diet for 17 weeks in HFD control groups significantly increased the weight of WAT compared to the normal. However, an 11 weeks treatment with 18KHT01 and orlistat significantly reduced WAT weight (Figure 7A). Similarly, increased liver weight in the HFD control group was significantly reduced with the treatment of 18KHT01 in a dose-dependent manner (Figure 7B). It has been accepted that hypertrophy of adipocytes occurs in high-fat diet-induced obesity (Hariri and Thibault, 2010). H&E staining analysis of WAT in this experiment also revealed the incidence of hypertrophy of adipocytes in the HFD control group. In addition, crown-like structures were observed in WAT indicating the development of obesity-induced local inflammation. Treatment with 18KHT01 ameliorated high-fat diet-induced inflammation and reduced the size of adipocytes as well (Figure 8). Thus, the mean area and Feret’s diameter of adipocytes were significantly reduced while adipocytes number were increased in 18KHT01 treated groups (Supplementary Table S8). The histological observation of liver tissue revealed that the accumulation of an enormous amount of lipid into the hepatocytes in the HFD control group indicating the development of liver steatosis. Treatment of 18KHT01 was found to be significantly effective in alleviating lipid accumulation in the liver (Figure 8). The results suggested that the 18KHT01 might be potential in the management of non-alcoholic fatty liver disease.

Comparison of effectiveness among 18KHT01 and its ingredients for preventing lipid accumulation in liver and WAT exhibited the 18KHT01 as the most effective in the reduction of WAT weight (Supplementary Table S9). Thus, the sizes of adipocytes were observed to be reduced to a large extent in the 18KHT01 among all the HFD fed groups (Supplementary Figure S7).

### 18KHT01 Synergistically Reduced Toxicity of Individual Plant Ingredients

In the synergistic anti-obesity study, the animals from each group were carefully monitored for the development of any sign of toxicity. One mouse from the *Q. acutissima* (QA, 150 mg/kg) group and three mice from *G. thunbergii* (GT, 150 mg/kg) were observed with lethal toxicity. No any treatment-related signs of toxicity were observed in the rest of the mice from QA and GT groups, or in the remaining groups. The summary of the clinical observations is presented in Supplementary Table S10. Systemic toxicity from the treated samples was further evaluated by performing liver and kidney function tests, hematological analysis, and histological observation of visceral organs (kidney and spleen). The liver function test revealed that the significantly elevated AST and ALT activities due to the HFD feeding were more exacerbated with GT, suggesting the death of mice might be because of liver damage. However, 18KHT01 alleviated each test parameter (Supplementary Table S11). Histological overview of the kidney tissue showed that a mild infiltration of macrophages and enlarge of Bowman’s space of glomeruli in CS and GT treated groups. These signs were observed to large extent in the QA treated group indicating a moderate renal inflammatory response. However, 18KHT01 ameliorated these histological alterations in the kidney (Supplementary Figure S9). A large extent of red pulp sinusoidal dilations was observed in HFD control and GT but not in other groups (Supplementary Figure S10). The hematological analysis revealed that the extent of neutrophil counts and hemoglobin levels were significantly reduced in CS, QA, and GT treated mice (Supplementary Table S12). In addition, QA and GT treated groups showed drastic high levels of platelet related parameters such as platelet counts (PLT), mean platelet volume (MPV), platelet distribution width (PDW), and plateletcrit (PCT) indicating systemic inflammation, and/or tissue damage (Bleeker and Hogan, 2011). The overall hematological data revealed that there were no treatment-related changes on blood parameters were observed in the 18KHT01 group, but the levels of some critical parameters were altered with the treatment of CS, QA, and GT, suggesting a significant synergistic effect of 18KHT01 on reducing toxic responses of the ingredients. The preliminary acute and sub-acute toxicity studies also showed the 18KHT01 as a safe herbal formulation in mice below 500 mg/kg/day for enduring application.

## Discussion

Obesity, caused by the over-deposition of lipids into WAT, is perceived as a global health risk in present days. Multiple metabolic maladies such as diabetes, cardiovascular diseases, fatty liver disease, and even certain cancers are discussed as complications of obesity (Baboota et al., 2013; Ananthakumar et al., 2020). However, obesity can be treated or prevented by assimilating certain interventions such as lifestyle-based (diet, exercise, and behavior therapy) and medical or surgical (pharmacotherapy or bariatric surgery) (Sengupta et al., 2012). Several pharmaceutical therapies have been recommended for the treatment of obesity. But, unexpected adverse effects by most of the anti-obesity drugs create controversies in using such therapies (Kang and Park, 2012). Compared to synthetic medicines, therapies based on natural resources are considered safe and more acceptable (Sengupta et al., 2012). A variety of herbal medicines or natural products including crude extracts, isolated compounds, and/or herbal formulations have been widely used for weight loss therapy (Yun, 2010). Several clinical studies demonstrated the anti-obesity activity of herbal formulation (Kamali et al., 2012; Lenon et al., 2012; Sengupta et al., 2012; Stern et al., 2013). The rationale behind using herbal formulation is to achieve polyherbalism or herbal synergy. The herb-herb interaction phenomenon can be a synergy when the combination produces greater effect than sum of the individual effect. In addition, synergy implies reduction of adverse effects of individual constituents (Zhou et al., 2016).

In the present study, an herbal formula (18KHT01) was developed by mixing a specific proportion of *Q. acutissima* (QA), *G. thunbergii* (GT), *C. sinensis* (CS), and *C. limon* (lemon juice). The anti-obesity prospective of these ingredients was described individually in the previous literatures. Green tea, a major component of 18KHT01 was widely studied previously in animal or clinical trials for its anti-obesity activity and it showed potential effect (Chantre and Lairon, 2002; Shimotoyodome et al., 2005; Choi et al., 2016; Muenzner et al., 2016). Few studies demonstrated the anti-obesity activity of *G. thunbergii* and *Q. acutissima* in overweight rodents as well (Sung et al., 2011; Kim et al., 2014; Hwang et al., 2017). As per the best of author’s investigations, there are very few or no studies conducted to evaluate the effect of *C. limon* fruit juice on obesity, but some studies established a role of other citrus fruits on the management of obesity (Nakajima et al., 2014; Montalbano et al., 2019; Carota et al., 2021). In this research work, 18KHT01 was developed and studied the synergistic effect of these herbal ingredients for the treatment of obesity by *in vitro* and *in vivo* methods.

Chronic inflammation like obesity is one of the major causes of systemic oxidative stress in the body (Liu et al., 2012). Obesity-associated oxidative stresses are involved in the development of obesity-induced disorders such as insulin resistance, hypertension, asthma, etc. (Manna and Jain, 2015). In this case, a natural anti-obesity remedy having antioxidant potentials could be a promising solution for protecting such obesity-associated oxidative stress and so its comorbidities. The potential and synergistic antioxidant activity in both the DPPH and ABTS model suggesting the 18KHT01 could be a better alternative for the prevention and protection of obesity and related complications. EGCG, a major active compound of 18KHT01, attenuates diet-induced obesity by enhancing fat oxidation (Klaus et al., 2005). At the initial phase of the development of obesity, differentiation of adipocytes is crucial onset (Gregoire, 2001). As obesity is aggravated by over-differentiation of the adipocytes, the inhibition of adipogenesis is considered a primary measure for the treatment of obesity (Jakab et al., 2021). The effects of 18KHT01 on 3T3-L1 adipocytes were determined to set a strategy for its anti-obesity activity. The results revealed that 18KHT01 treatment at Day 0, but not at Day 2, significantly reduced lipid accumulation in 3T3-L1 adipocytes (Figures 2A,B). This observation may suggest that 18KHT01 only plays an inhibitory role at the initial phase of adipogenesis. In the synergy evaluation experiment, an anti-adipogenic activity of 80 μg/ml of 18KHT01 and the same dose of CS were observed to be comparable, showed lipid inhibition about 30% (Figure 2C). But, 43.90 μg/ml of CS (equivalent amount of CS in 80 μg/ml of 18KHT01) exhibited only 7.72 ± 4.60% of lipid inhibition (Table 3). This result indicated the activity possessed by 18KHT01 was not only as a result of presence of CS. A previous study also revealed that decaffeinated green tea extract with a dose of 2 mg/ml did not decrease lipid accumulation in 3T3-L1, but higher doses were significantly active (Yang et al., 2014). In addition, the inhibition of lipid production by separately treated 18KHT01 was higher than the cumulative lipid inhibition by individually treated 18KHT01 ingredients (Table 3). These evidences proved a gained of admirable synergy for the anti-adipogenic activity of 18KHT01.

Adipogenesis is a multistep signaling process, which is initiated with the activation of specific transcription factors such as PPARγ, C/EBPα, and SREBP-1c via several biological pathways. The process of adipogenesis is maintained thereafter with the action of other adipogenic factors such as aP2, FAS, and LPL (Sarjeant and Stephens, 2012; Moseti et al., 2016). In addition, leptin, an adipokine secreted mainly by adipocytes, is also considered to induce as well as promote adipogenesis in 3T3-L1 adipocytes (Palhinha et al., 2019). Therefore, down-regulations of these adipogenic factors may restrict initial as well as terminal differentiation, leading to an inhibition of lipid accumulation in adipocytes (Jakab et al., 2021). In this study, down-regulation of gene expression of PPARγ, C/EBPα, SREBP-1c, aP2, FAS, LPL, and leptin in 3T3-L1 adipocytes by 18KHT01 (Figure 3) suggested the potential effect of 18KHT01 on preventing adipogenesis, so prevent obesity.

The exciting outcomes from *in vitro* experiments encouraged us to conduct an *in vivo* anti-obesity study. Diet-induced obese (DIO) C57BL/6J mice model was used to evaluate the curative anti-obesity effect of 18KHT01. The DIO mouse model mimics human obesity and may hypothesize if the 18KHT01 is effective for obese people. It is well-described in a lot of studies that the long-term feeding of HFD induces obesity in rodents (Sclafani, 1984; Surwit et al., 1988; Hariri and Thibault, 2010). The HFD induced obesity may be characterized by an increase in body weight, weight gain, FER, body fat content, and hyperglycemia (Ikemoto et al., 1996; Hariri and Thibault, 2010; Yang et al., 2017). In this study, 6 weeks of the feeding of HFD significantly increased body weight, weight gain, FER, and fasting blood glucose level in C57BL/6J mice indicating successful development of the DIO mice model. Obesity can be put into a category of incurable chronic disease (Kang and Park, 2012). Thus, restriction in body weight gain without adverse effects is considered an effective therapy in the management of obesity. The body weight and weight gains in DIO mice were significantly reduced by 18KHT01 during 11 weeks of drug treatment period even continuing the HFD feeding (Figures 4A,C). In 18KHT01 treated groups, the stool lipid excretion was not significantly differed from the HFD control group (data not shown) but the FER was significantly lowered. These results suggested that the 18KHT01 may exert systemic actions for its anti-obesity activity.

Hyperglycemia or diabetes is one of the major complications of obesity. Previous studies also revealed that the HFD fed mice developed hyperglycemia as well as insulin resistance (Ikemoto et al., 1996; Hariri and Thibault, 2010). The significantly decreased level of blood glucose in the 18KHT01 groups compared to the HFD control group indicates that the 18KHT01 might have a protecting effect against insulin resistance and related complications. The systemic insulin resistance had quantified in the term of HOMA-IR index (Rossmeisl et al., 2003). In the current study, a significantly high value of HOMA-IR in the HFD control group due to the elevation of blood glucose level and insulin concentration indicated a development of insulin resistance. However, the treatment of 18KHT01 significantly reduced HOMA-IR index compared to the HFD control (Figure 5C). In addition, only 18KHT01 treated group showed significantly lowered serum insulin concentration and HOMA-IR index in the synergy evaluation animal experiment (Figures 5D,E). Hence, the results suggested that the 18KHT01 may have high potential for the treatment of obesity induced diabetes and insulin resistance.

Previous studies exposed the scientific evidence that the dietary high fat may lead to liver steatosis which is associated with obesity and liver dysfunction (Panchal et al., 2011). In this study, 17 weeks exposure of high-fat diet led to an accumulation of massive lipid globules in the liver indicating the development of liver steatosis in the HFD control group. The accumulation of fat into liver cells caused a significant increase in liver weight (Figure 7B). However, 18KHT01 was effective in protecting from liver steatosis by reducing lipid accumulation and so significant reduction of liver weight (Figures 7B, 8). Similarly, obesity is primarily associated with the deposition of lipids into WAT (Vazquez-Vela et al., 2008). The accumulated visceral fat in epididymal adipocytes due to HFD feeding was significantly reduced with the treatment of 18KHT01 (Figures 7A, 8) indicating the potent anti-obesity property of 18KHT01 in obese C57BL/6J mice.

Obesity is accompanying with dyslipidemia characterized by elevation of serum cholesterols and triglyceride levels. Dyslipidemia contributes a major pathophysiological role in cardiovascular diseases, atherosclerosis, stroke, and diabetes (Mooradian, 2009; Qi et al., 2015). Treatment with 18KHT01 exhibited a significant reduction of TC, TG, and LDL/VLDL compared to the HFD control group (Figure 6), indicating 18KHT01 as effective in ameliorating dyslipidemia and associated comorbidities. In the synergy evaluation animal experiment, only 18KHT01 was found to be effective in reducing serum TG levels. Inversely, CS was inactive towards serum TG, whereas QA and GT elevated TG levels at 150 mg/kg dose (Figure 6F). This result indicates a strong synergy of the ingredients in 18KHT01 for reducing serum TG levels. Consistent with the result of this study, a report showed that 0.25% of green tea extract was not effective in reducing plasma TG level, but significantly reduced other plasma lipids as well as hepatic TG concentration in obese mice (Choi et al., 2016). In contrast to this result, a previous study demonstrated that 200 mg/kg of *Q. acutissima* and 400 mg/kg of *G. thunbergii* showed a significant reduction of serum TG level in high-fat diet-fed obese C57/BL6 mice (Sung et al., 2011; Hwang et al., 2017).

The formulation 18KHT01 was found to reduce systemic adverse effects produced by the individual ingredients. Treatment of 300 mg/kg of both QA and GT showed a lethal toxic effect in animals. Previous studies demonstrated that consumption of different parts including acorns of *Quercus* species caused renal toxicity in lamb and horses. A high dose of acorn also caused toxic symptoms with severe nephrosis, gastroenteritis, and even death in cattle (Chamorro et al., 2013; Smith et al., 2015). Injection of acorn extracted hydrolyzable tannin in mice, rabbits, and calf caused mortality by liver and kidney damage (Clarke and Cotchin, 1956). Hence, the mortality in one mice of the QA group might be because of kidney damage. This hypothesis was also supported by histological observation of the kidney (Supplementary Figure S9). However, some publications with the administration of ethanol extract of *Q. acutissima* acorn below 200 mg/kg/day dose in mice did not report any toxicological responses (Moon et al., 2012; Hwang et al., 2017). There are no published reports on *Quercus* acorn toxicity in humans. Three of five mice from the GT group have died of toxicity. Mice were observed with severe toxic symptoms such as drowsiness, sedation, laborious breathing before death (Supplementary Table S10). A high discoloration of the liver was observed in the necropsy of a euthanized mouse. This observation advocates that the mortality in the GT group might be because of liver damage. The increased in the extent of AST and ALT activities in the GT group also indicated the development of hepatic injury (Supplementary Table S11). However, some of the previous publications did not report toxic responses even with the administration of 400 mg/kg/day of 70% ethanol extract of *G. thunbergii* for 6.5 weeks (Sung et al., 2011). There were no observed any toxicological responses in 18KHT01, and CS treatment groups. However, there are many evidences that the consumption of a high amount of green tea caused undesirable effects. Administration of high dose of green tea polyphenol induced renal and hepatic abnormality in mice (Inoue et al., 2011). Numerous case reports specified a hepatotoxic effect of a high dose of green tea (Mazzanti et al., 2009; Patel et al., 2013). However, a lower dose of green tea and green tea preparations are safe and effective in various ailments (Sharangi, 2009). As the 18KHT01 is composed of partial amounts of the QA, GT, and CS, the toxic effects observed with the high dose of these individual ingredients may be eliminated with the administration as the form of 18KHT01 formulation.

The difference in the level of hematological parameters in 18KHT01 ingredient-treated groups indicated toxicological responses produced by them. The CS, QA, and GT groups possessed a significant reduction of neutrophil count and hemoglobin level, but these parameters were in baseline level in the 18KHT01 group (Supplementary Table S12). A low level of neutrophil count is associated with bone marrow disorders, chronic inflammations, vitamin B12, and folic acid deficiency, aplastic anemia, and numerous other possible factors (Newburger and Dale, 2013). As green tea interferes with the absorption of folic acid (Alemdaroglu et al., 2008), the lower count of neutrophils in the CS group might be due to the development of folic acid deficiency. A previous study also revealed the green tea supplement decreased monocyte and neutrophil counts (Zak and Pokora, 2017). Green tea is also considered to interfere with iron absorption (Hamdaoui et al., 2003; Fan, 2016). As iron is an essential component of hemoglobin, the low level of hemoglobin in the CS group might be due to iron deficiency. The abundant amount of tannins present in CS, QA, and GT may also be responsible for the iron deficiency and so low hemoglobin level (Delimont et al., 2017). The QA and GT groups exhibited drastically increased levels of platelets count. The high platelets count in mice might associate with systemic inflammation, tissue damage, and/or iron deficiency anemia (Bleeker and Hogan, 2011). All of the critical blood parameters in the 18KHT01 group were observed to be at baseline as in the normal groups. Hence, the overall hematological investigation is the strong evidence showing synergistic efficacy of 18KHT01 on reducing the systemic toxicity of the individual ingredients.

## Conclusion

From all the observations, it can be concluded that the high-fat diet-induced obesity was potentially controlled in mice by the administration of 18KHT01. In most of the experiments, the 18KHT01 (150 mg/kg) exhibited better outcomes than those by the standard orlistat (10 mg/kg). The 18KHT01 might show anti-obesity activity through the collective effects of inhibition of adipocyte differentiation; demotion of serum lipid levels; and inhibition of fatty acid accumulation in tissues. In addition, 18KHT01 showed potential antioxidant activity. The synergy evaluation study revealed a small amount of the 18KHT01 ingredients incorporated as a formulation showed potent anti-obesity activity, which was better than the effects achieved by higher doses of the individual ingredients. Moreover, a combination of the ingredients as 18KHT01 reduced adverse effects bared by individual ingredients. Thus, synergy on 18KHT01 was developed by improving efficacy and by reducing the toxicity of the ingredients. Therefore, consumption of the novel polyherbal formulation, 18KHT01, may be effective for the management of human obesity and its comorbidities such as insulin resistance and oxidative stress. However, a detailed molecular mechanism of anti-obesity activity of 18KHT01 is yet to be investigated.

## Data Availability

The original contributions presented in the study are included in the article/Supplementary Material, further inquiries can be directed to the corresponding author.

## Abbreviations

DIO, diet-induced obesity; CS, *Camellia sinensis*; QA, *Quercus acutissima*; GT, *Geranium thunbergii*; PPARγ, peroxisome proliferator-activated receptor gamma; C/EBPα, CAAT/enhancer binding protein alpha; SREBP-1c, sterol response element binding protein- 1c; LPL, lipoprotein lipase; FAS, fatty acid synthase; FABP4/aP2, adipocyte-specific lipid binding protein; EGCG, (-)-epigallocatechin-3-gallate; EGC, (-)-epigallocatechin; ECG, (-)-epicatechin-3-gallate; EC, (-)-epicatechin; UPLC, Ultra Performance Liquid Chromatography; DPPH, 1, 1 diphenyl-2-picrylhydrazyl; ABTS, 2,2'-azino-bis(3-ethylbenzothiazoline-6-sulfonic acid; PBS, phosphate buffer saline; IC_50_; inhibitory concentration 50%; CI, combination index; DMEM, Dulbecco’s modified eagle medium; NCS, new-born calf serum; IBMX, 3-Isobutyl-1-methylxanthine; MTT, Thiazolyl blue tetrazolium bromide; LDH, lactate dehydrogenase; ORO, oil red O; PCR, polymerase chain reaction; HFD; high-fat diet; FER, food efficiency ratio; CMC, carboxymethyl cellulose; HOMA-IR, homeostasis model assessment of insulin resistance; TC, total cholesterol; LDL/VLDL, low/ very low-density lipoprotein cholesterol; HDL, high density lipoprotein; TG, triglycerides; ALT, alanine aminotransferase; AST, aspartate aminotransferase; AI, Atherogenic index; WAT, white adipose tissue.
