# Supplementary material for: 18KHT01, a Potent Anti-Obesity Polyherbal Formulation
Source: Front Pharmacol. 2021 Dec 17;12:807081. doi: 10.3389/fphar.2021.807081 (PMC8719591; doi:10.3389/fphar.2021.807081)
Supplement: Supplementary file 1 [file DataSheet1.docx]

Supplementary Material

**18KHT01, a potent anti-obesity polyherbal formulation**

Prakash Raj Pandeya^1, 2^, Ramakanta Lamichhane^1^, Gopal Lamichhane^1^, Kyung-Hee Lee^1^, Hyeong Kyu Lee^3^, Su-Jin Rhee^4^, Hyun-Ju Jung^1*^

^1^Department of Oriental Pharmacy and Wonkwang-Oriental Medicines Research Institute, Wonkwang University, Sinyong-Dong, Iksan, 570-749, South Korea

^2^Bio-Safety Research Institute, Jeonbuk National University, Iksan, 570-752, South Korea

^3^Natural Medicine Research Center, Korea Research Institute of Bioscience and Biotechnology (KRIBB) Yeongudanji-ro 30, Ochang-eup, Cheongju-si 28116, South Korea

^4^Department of Pharmacy, Wonkwang University, Sinyong-Dong, Iksan, 570-749, South Korea

*** Correspondence:**Prof. Hyun-Ju Jung, Ph.D. (HJJ)
hyun104@wku.ac.kr

**Experimental materials:**

**Herbal ingredients of 18KHT01**

Acorn jelly powder of *Quercus acutissima* (QA) was purchased from a food company Choroc Maeul (Seoul, Korea). Dry leaf buds of *Camellia sinensis* (CS – green tea) and dry aerial parts of *Geranium thunbergii* (GT) were purchased from a medicinal herb store Begjangseng (Iksan, Korea). Fruits of *Citrus limon* were purchased from a local market.

**Solvents, chemical Reagents, and chromatographic supplies**

HPLC grade ethanol, methanol, acetonitrile, and isopropanol were purchased from SK Chemicals (Seongnam, Korea). ECG, EC, and ellagic acid were purchased from Sigma-Aldrich Inc. (St. Louis, MO, USA). Caffeine and EGCG were purchased from Fujifilm Waka pure chemicals Co. (Osaka, Japan). Corilagin was previously isolated in the lab from *G. thunbergii*. A chromatographic column (Halo 90 Å RP-Amide (2 µm, 2.1 x 150 mm) was purchased from Advanced Materials Technology (Munich, Germany). DPPH (1, 1 diphenyl-2-picrylhydrazyl) reagent was obtained from Wako Pure Chemical Industries (Tokyo, Japan). ABTS reagent (2, 2′-azino-bis (3- ethylbenzothiazoline-6-sulfonic acid) diammonium salt was purchased from Sigma-Aldrich (St. Louis, MO, USA). Potassium persulfate was purchased from Daejung Chemicals (Jeongwang-dong, Korea).

**Cell culture and bioassay reagents**

Mouse embryonic fibroblast, adipose like cell line (3T3-L1 pre-adipocytes) was obtained from American Type Culture Collection (ATCC). Dulbecco's Modified Eagle Medium (DMEM), newborn calf serum (NCS) and fetal bovine serum (FBS) were obtained from Gibco, USA. 3-Isobutyl-1-methylxanthine (IBMX), dexamethasone, insulin, 10 % formalin, isopropanol and Oil Red O (ORO) were purchased from Sigma-Aldrich (St. Louis, MO, USA). Thiazolyl blue tetrazolium bromide (MTT) was purchased from Alfa Aesar, England. Dimethyl sulfoxide (DMSO) was obtained from Junsei, Japan. Qiazol lysis reagent was purchased from Qiagen Sciences (Maryland, USA). High capacity RNA-to-cDNA kit and power SYBR-Green PCR master mix was obtained from Applied Biosystems, UK. Colorimetric Assay Kits for LDH (Catalog # K313-500); Total Cholesterol (Catalog # K603-100); HDL and LDL/VLDL (Catalog # K613-100); and Triglyceride (Catalog # K622-100); Creatinine (Catalog # K625-100); Bilirubin (Catalog # K553-100); ALT activity (Catalog # K752-100); and AST activity (Catalog #K753-100) were purchased from BioVision (BioVision, Milpitas, USA). Mouse Insulin ELISA Kit (AKRIN-011T) was purchased from Shibayagi Co., Japan.

**Instrumental**

Extract solutions were concentrated using rotary evaporator (Tokyo rikakikai Co., ltd., Japan) and were freeze dried on Lyophilizer (IIShin Lab Co., Ltd., Korea). Phytochemical analysis was performed using ultra performance liquid chromatography (UPLC) system (Agilent Technologies, Santa Clara, CA, US) consisting of a G4220A 1290 Infinity Binary pump, a G4226A 1290 auto-sampler and a G4242A 1290 DAD detector. PCR was carries by using StepOnePlus Real-Time PCR system from Applied Biosystems Inc. (Marsiling Industrial Estate Road 3, Singapore). Absorbance was measured using micro-plate reader (Spectra max 190, USA). Micrographs were captured using EVOS XL core light microscope (Life Technologies, Bothel, WA, USA). Blood glucose was measured using one-touch blood glucose monitoring system (CareSens ®N, i-sens, Korea).

**Supplemental Figures**

**Figure S1**


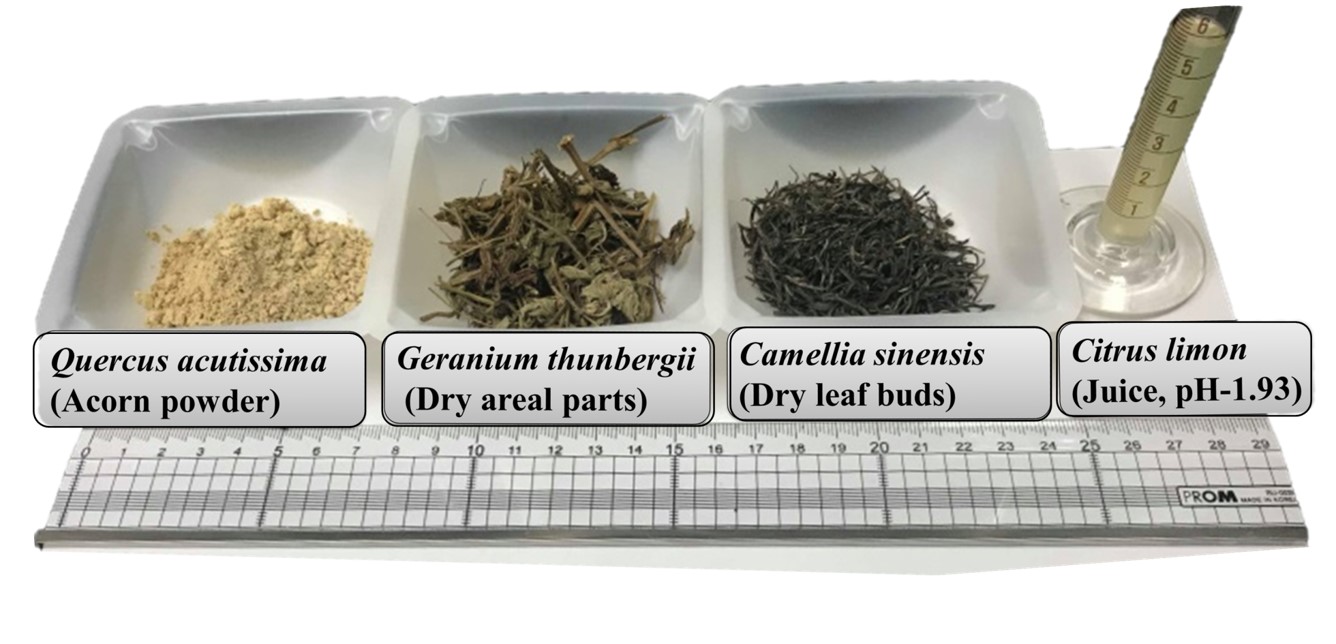
**Figure S1: Ingredients of 18KHT01.**

**Figure S2**


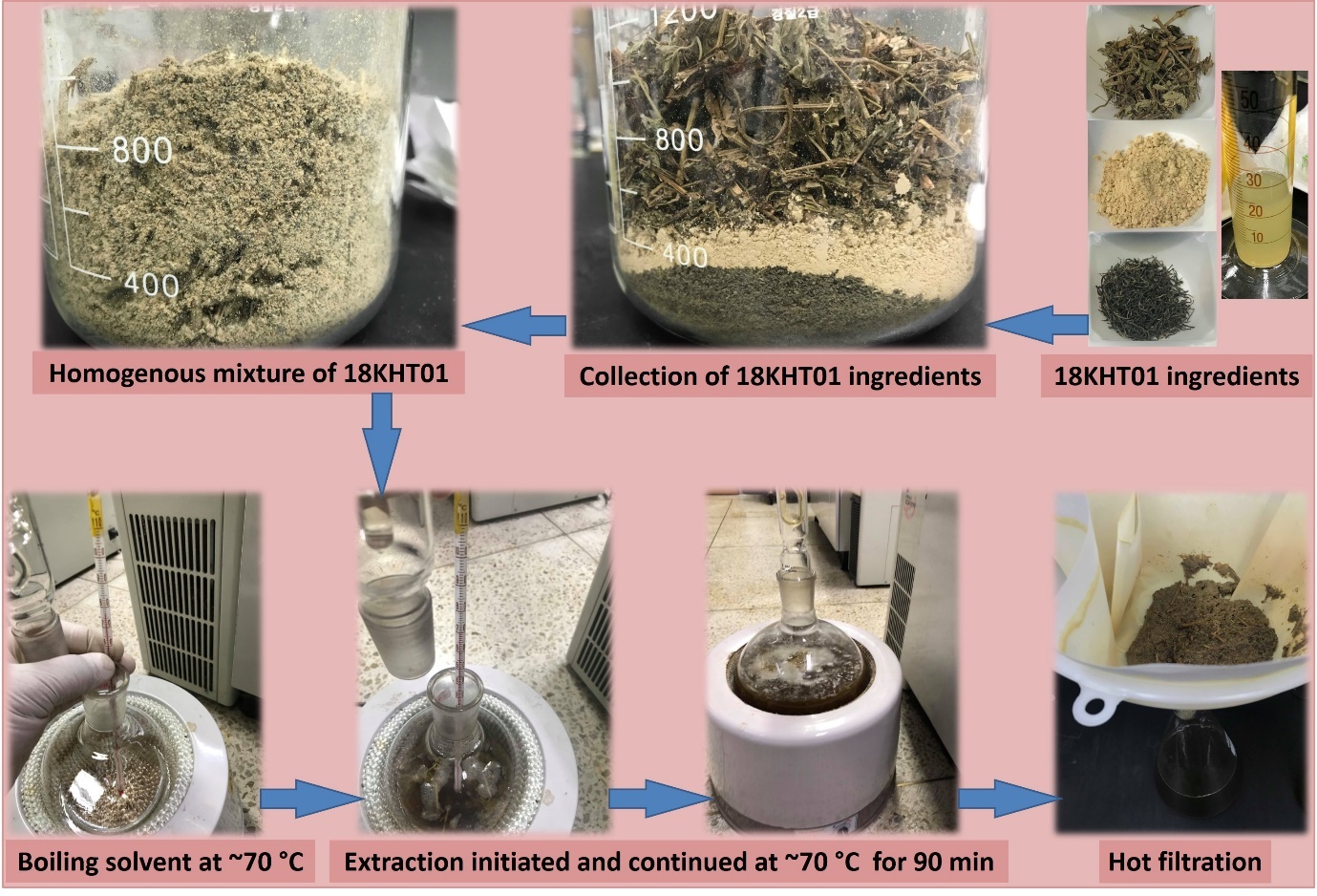


**Figure S2: Schematic diagram of extraction procedure of 18KHT01.** The ingredients were ground, weighed, collected, and mixed homogeneously. Before extraction, the solvent (40% ethanol/distilled water) was subjected to boil to reach 70 ᴼC temperature. Once the temperature become stable, the homogeneous raw formulation was mixed with hot solvent and refluxed at 70 ᴼC for 90 min at a constant temperature. After completing the extraction, the hot extract was immediately filtered and was subjected to evaporate in a vacuum evaporator at 42 ᴼC.

**Figure S3**


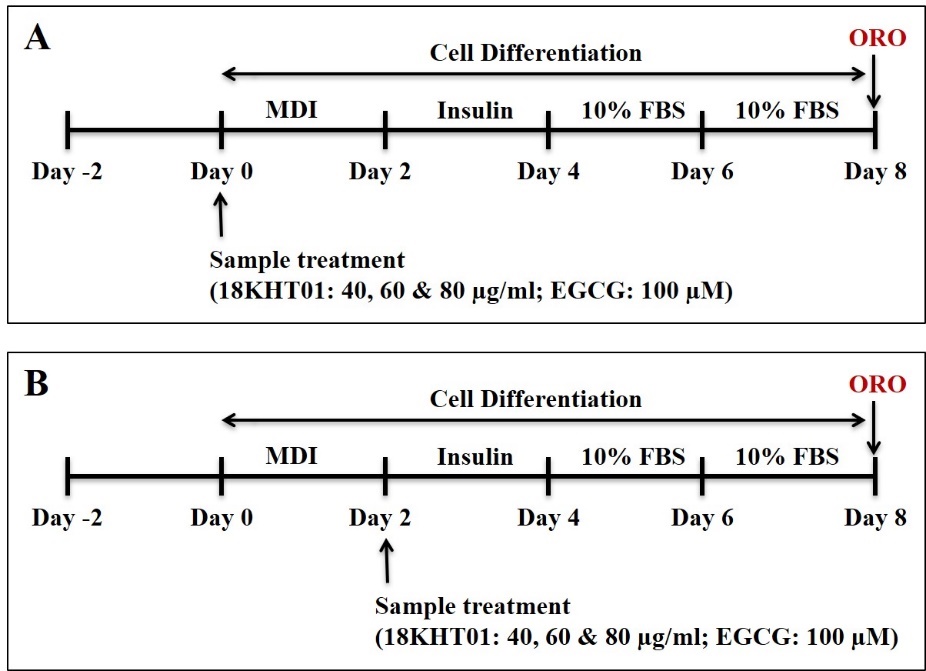


**Figure S3: Schematic diagram of cell differentiation and treatment of 18KHT01 at Day 0 (A), and Day 2 (B)**. Two days later of the cell confluence, adipocyte differentiation was induced by treating adipogenesis induction media (MDI) containing a cocktail of 3-Isobutyl-1-methylxanthine (IBMX- 0.5mM), Dexamethasone (1 µM), and insulin (5 μg/mL) in 10% FBS/DMEM. The MDI media was replaced by 5 μg/mL of insulin in 10% FBS/DMEM after two days of induction (at Day 2), for adipocyte maintenance. Thereafter the cells were replaced with 10% FBS two times in every 2 days (at Day 4 and Day 6). The 18KHT01 was treated separately at different stages (Day 0 and Day 2) of the cell differentiation to examine their effectiveness in different stages of adipogenesis. At the end of the experiments (at Day 8), the cells were washed with PBS once and fixed in 10% formalin solution.

**Figure S4**


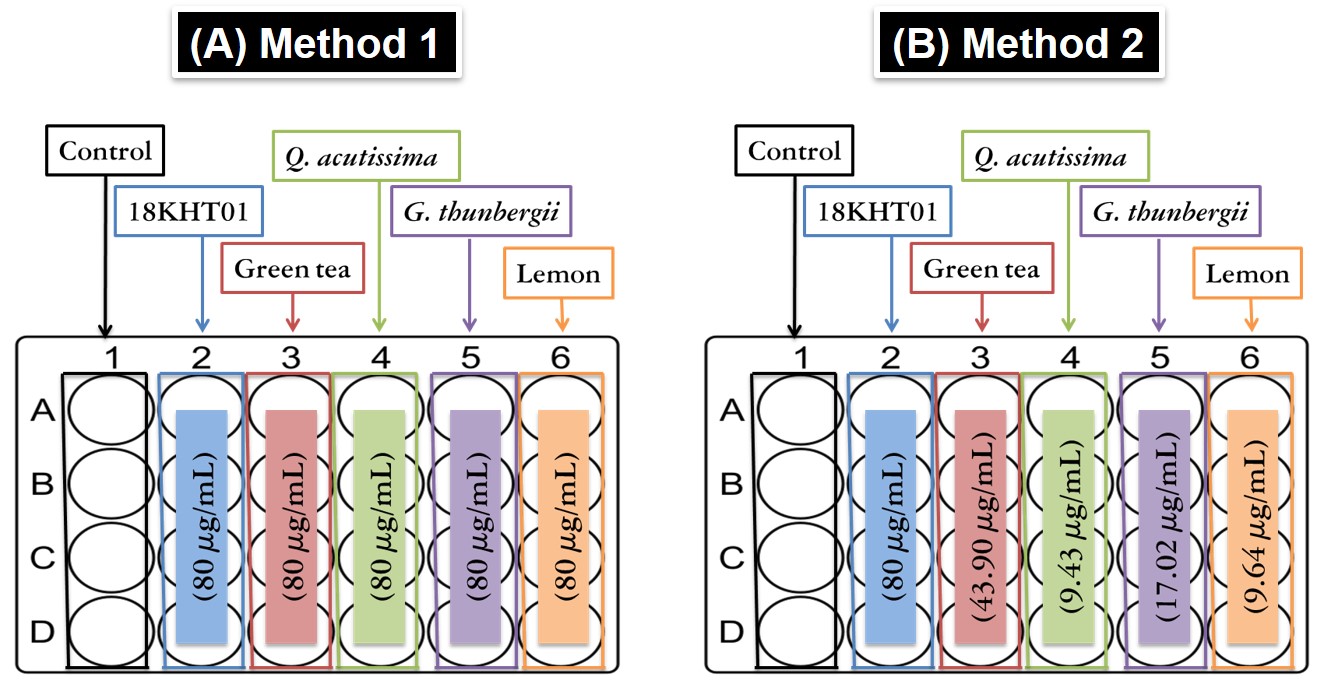


**Figure S4: Two methods of treatment of 18KHT01 and its ingredients to 3T3-L1 adipocytes for evaluating synergistic anti-adipogenic activity.** In method 1 (A), 18KHT01 and its all ingredients were treated at an equal concentration of 80 μg/mL, and activities were compared. In method 2 (B), an 80 μg/mL of 18KHT01 and equivalent amounts of ingredients present on the 80 μg/mL concentration of 18KHT01 were separately treated and cumulative activity by ingredients was compared with the activity bared by 18KHT01 alone.

**Figure S5**


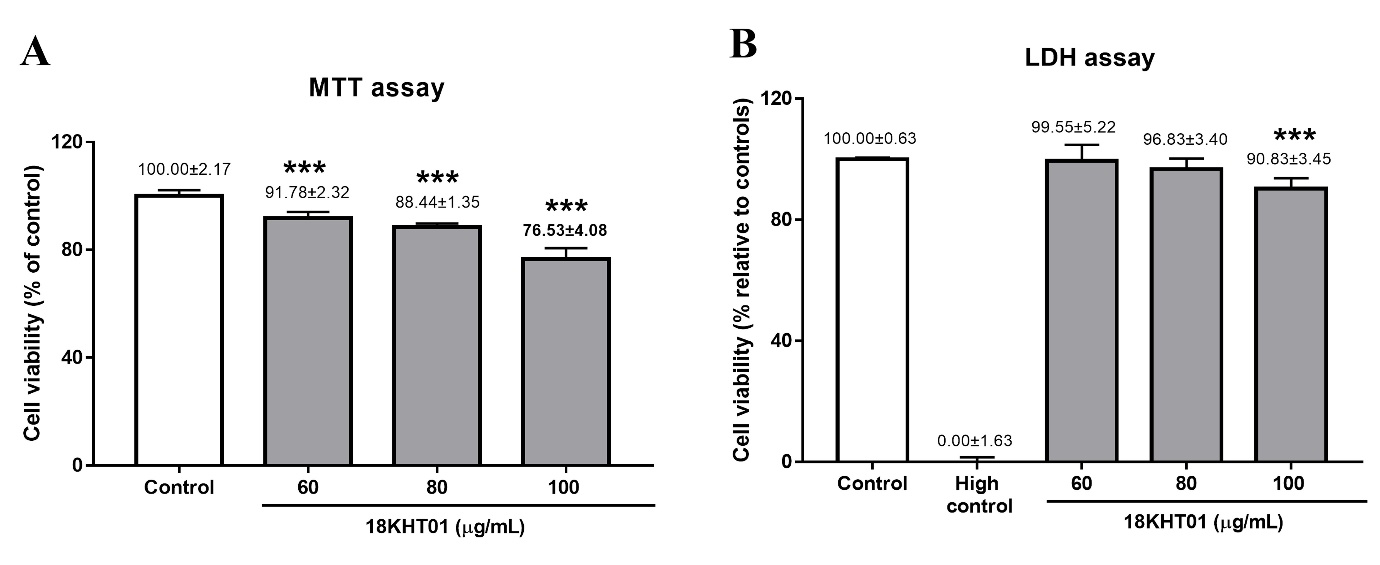


Figure S5: Effect of 18KHT01 on Cell viability of 3T3-L1 adipocytes. The 3T3-L1 cells were treated with different concentrations of 18KHT01 in two separate 48 well plates and the cell viability was assessed by (A) MTT assay and (B) LDH assay. The percentage of cell viability of samples was calculated as: MTT cell viability (%) = Abs_sample_/Abs_control_ × 100, and LDH cell viability (%) = 100-[ (Abs_sample_ – Abs_low control_)/( Abs_high control_ – Abs_low control_) × 100]. The cell viability assessed by MTT assay was observed to be lesser than that by LDH assay. These observations indicated the anti-proliferative effect of 18KHT01, where the proliferation of the 3T3-L1 cells was stopped without toxic effect. Statistical significance was calculated using one-way ANOVA followed by Dunnett's multiple comparisons test. Data are presented as mean ± SD (n=6) with significance ****P* < 0.001 vs. control.

**Figure S6**


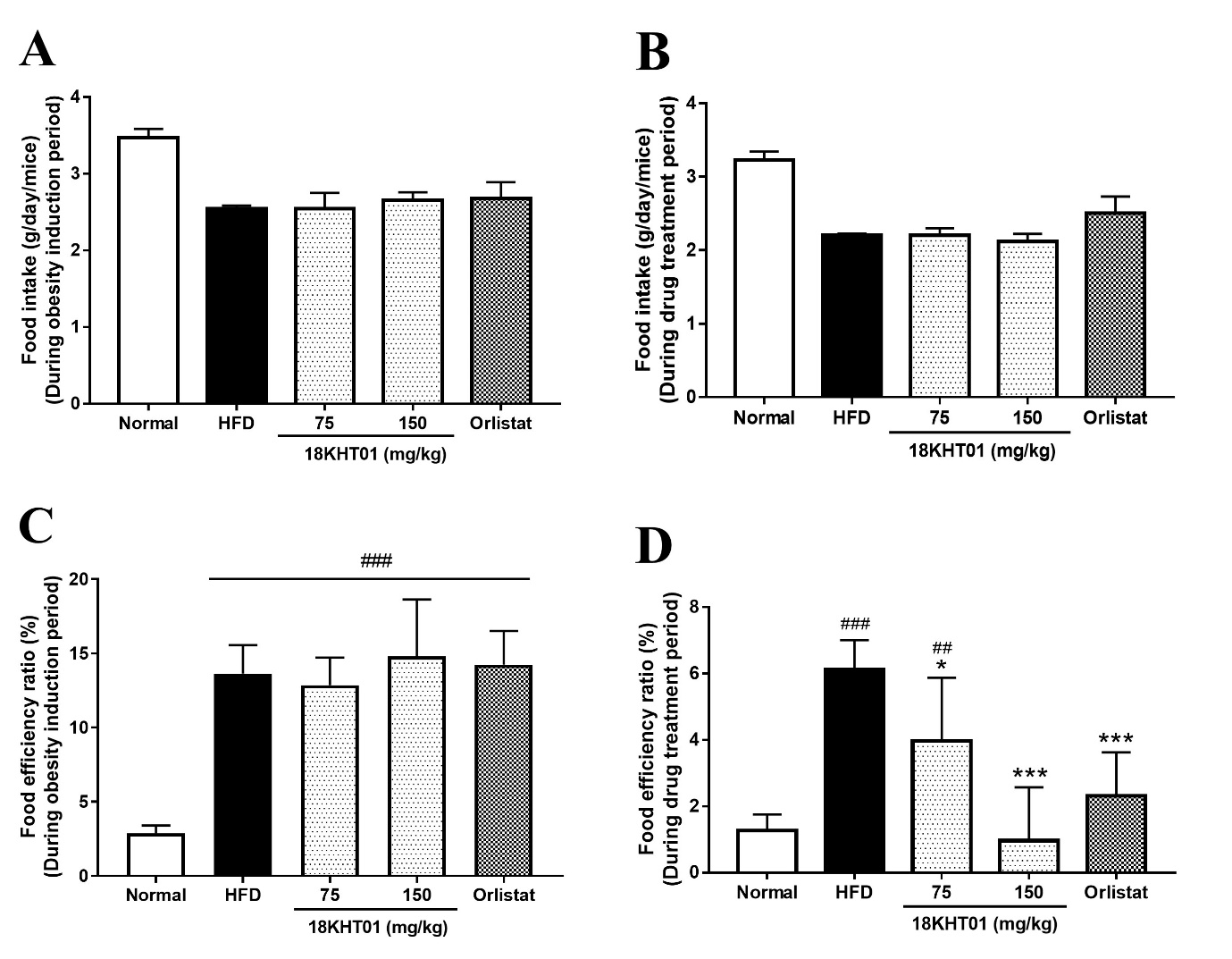


Figure S6: Effect of 18KHT01 on food intake and food efficiency ratio (FER). Food intake and body weight gains were measured every week. The food intakes (A) during the obesity induction period and (B) during the drug treatment period were separately presented. The food efficiency ratio (FER) was calculated as follows: FER% = gained body weight (g) × 100 / food intake (g). The FER (C) during 6 weeks of the obesity induction period and (D) during 11 weeks of the drug treatment period were separately calculated. Values of food efficiency ratios are expressed as mean ± standard deviation (n = 6). Statistical significance was calculated using one-way ANOVA followed by Dunnett's multiple comparisons test. Data are presented with significance ^##^*P* < 0.01, ^###^*P* < 0.001 vs. Normal; **P* < 0.05, ***P* < 0.01, ****P* < 0.001 vs. HFD control. Normal = Standard chow diet; HFD = high-fat diet control; Orlistat = orlistat (10 mg/kg).

**Figure S7**


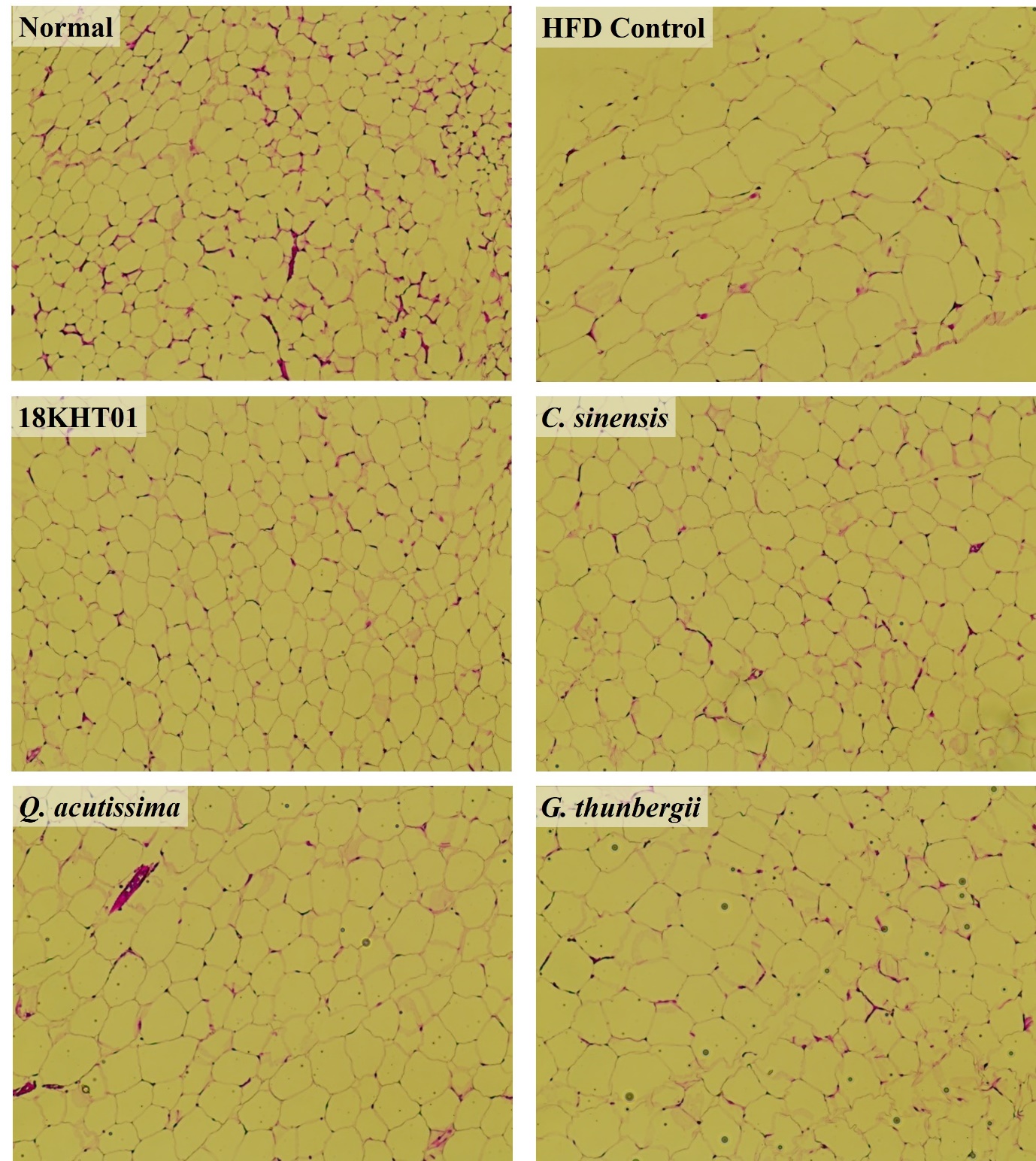


**Figure S7: Effect of 18KHT01 and its ingredients on white adipose tissue (WAT) histology.** Hematoxylin and eosin (H&E) staining was done to perform histological analysis. Pictures were captured at 20× magnification under a light microscope. Sizes of adipocytes were evaluated.

**Figure S8**


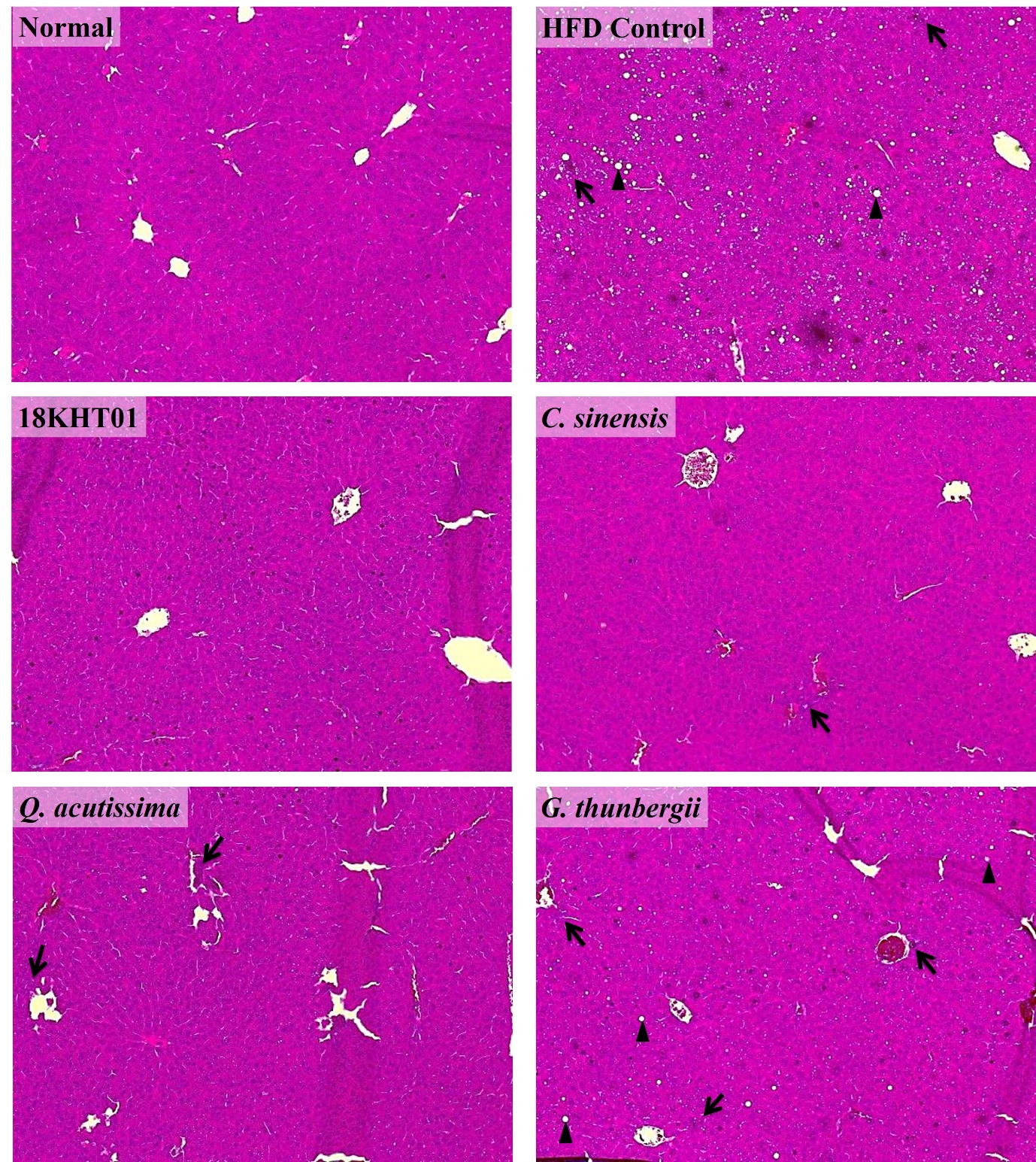


**Figure S8: Effect of 18KHT01 and its ingredients on liver histology.** Hematoxylin and eosin (H&E) staining was done to perform histological analysis. Pictures were captured at 20X magnification under a light microscope. Accumulation of lipid droplets (arrowhead) and macrophage infiltrations (arrow) are evaluated. Lipid droplets are more noticeable in HFD control and GT groups.

**Figure S9**


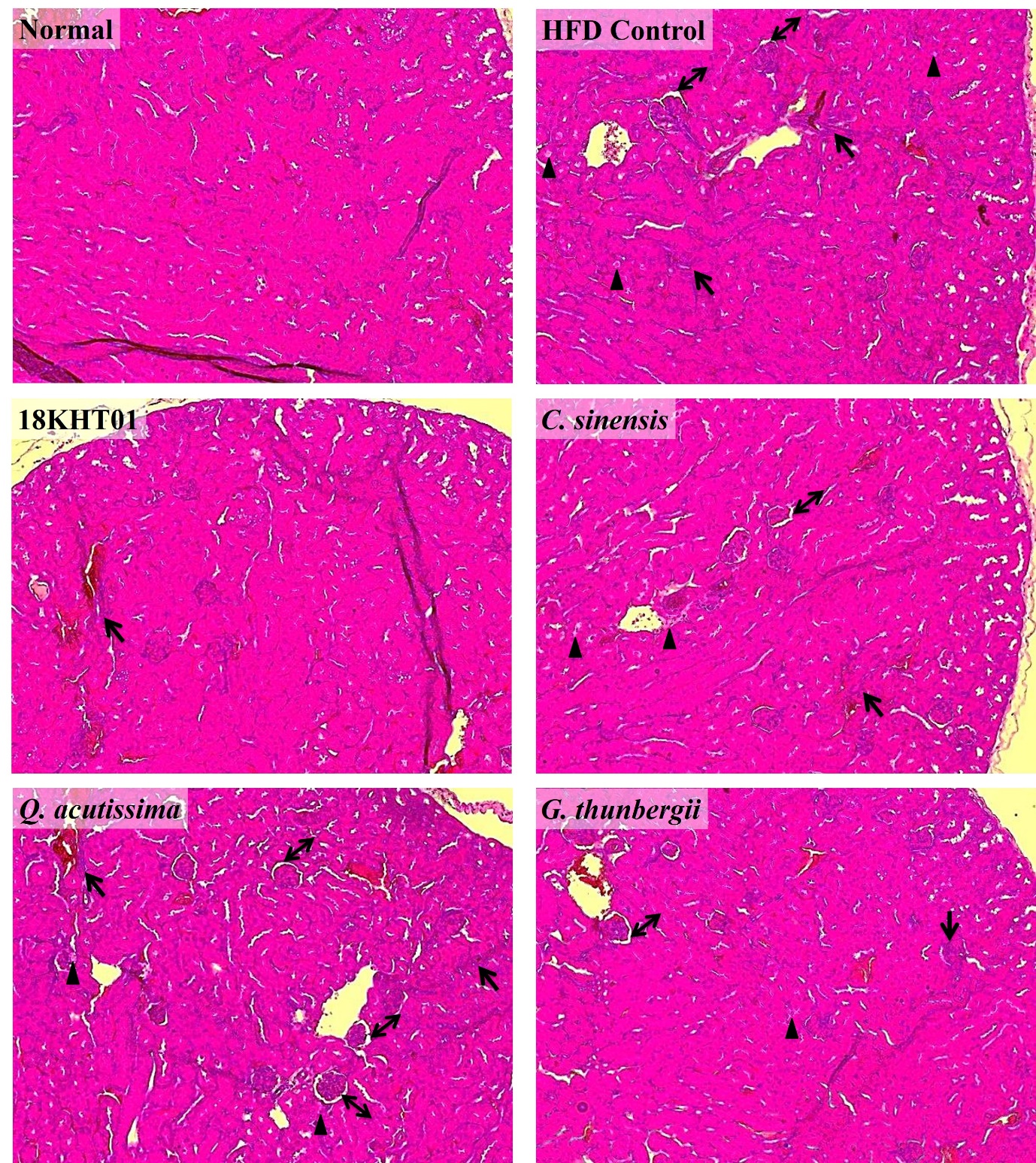


**Figure S9: Effect of 18KHT01 and its ingredients on kidney histology.** Hematoxylin and eosin (H&E) staining was done to perform histological analysis. Pictures were captured at 20X magnification under a light microscope. Glomerular and tubular lipid ambulation (arrowhead), expansion of Bowman’s space in capsule (double-headed arrow), and macrophage infiltration (single-headed arrow) are evaluated. Tubule-interstitial, as well as glomerular lipid accumulations, occur in the HFD control group, indicating the development of renal steatosis. Treatment of 18KHT01 inhibited renal lipid accumulation, whereas some extents of lipid accumulation were noticeable in CS, QA, and GT groups. Mild infiltration of macrophages and enlarge of Bowman’s space of glomeruli were also noticeable in HFD control as well as CS, QA, and GT treated groups compared to the normal group. Macrophage infiltration and enlargement of Bowman’s space were observed in the QA group to large extent, indicating a moderate renal inflammatory response. However, 18KHT01 ameliorated these histological alterations in the kidney, indicating synergistically better efficacy in reducing HFD induced renal steatosis and inflammation.

**Figure S10**


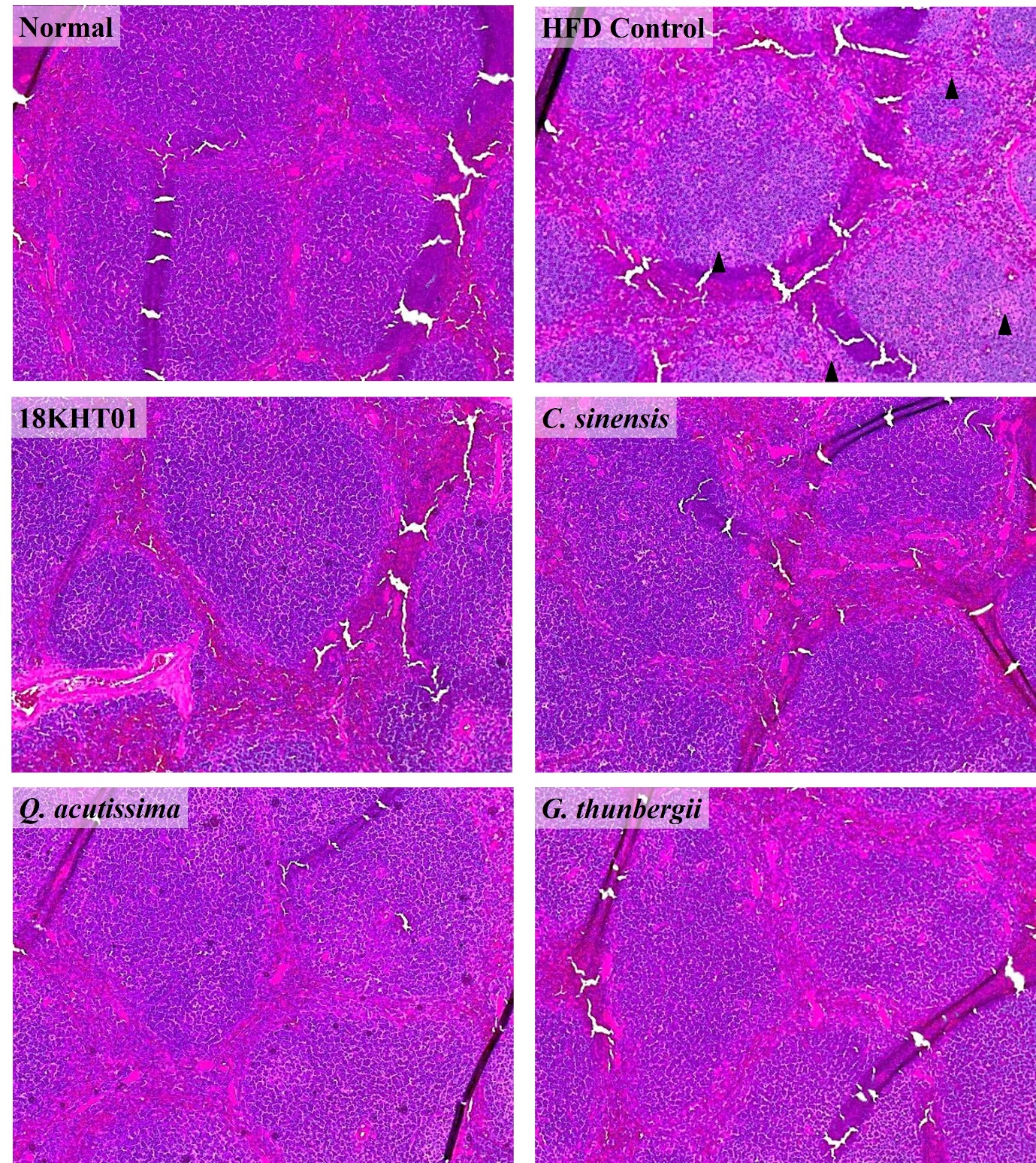


**Figure S10: Effect of 18KHT01 and its ingredients on spleen histology.** Hematoxylin and eosin (H&E) staining was done to perform histological analysis. Pictures were captured at 20X magnification under light microscope. Histological architectures of white and red pulp and lipid accumulations (arrowhead) were evaluated. Lipid accumulations into sinusoids are more noticeable in the HFD control group. A large extent of red pulp sinusoidal dilations was observed in HFD control and GT groups.

**Supplemental Tables**

Table S1. Yield value of the individual ingredients in 40% ethanol solvent and the composition of crude and 40% ethanol extract of 18KHT01

| **Ingredients** | **Crude composition of unit dose of 18KHT01** | | **Yield (%)** | **Composition of 18KHT01 40% ethanol extract based on individual yield value** | |
| --- | --- | --- | --- | --- | --- |
|  | Amount (g) | Amount (%) |  | Amount (g) | Amount (%) |
| *Q. acutissima* (QA) | 8.94 | 37.41 | 5.16 | 0.46 | 11.79 |
| *G. thunbergii* (GT) | 3.74 | 15.65 | 22.23 | 0.83 | 21.28 |
| *C. sinensis* (CS) | 5.63 | 23.56 | 38.03 | 2.14 | 54.87 |
| *C. limon* (Lemon) | 5.43 mL (~5.59 g) | 23.39 | 8.34 | 0.47 | 12.05 |

**Table S2: Chromatographic condition used for the phytochemical analysis of 18KHT01**

| System | Agilent 1290 UPLC | | | |
| --- | --- | --- | --- | --- |
| Column | Halo 90 Å RP-Amide (2 µm, 2.1 x 150 mm) | | | |
| Column temperature | 40 ᴼC | | | |
| Flow rate | 0.3 ml/min | | | |
| Injection volume | 2 µL | | | |
| Mobile phase | A: Acetonitrile | | B: 0.1% phosphoric acid/water | |
| **Method** | **Time (min)** | **A (%)** | | **B (%)** |
|  | 0 | 1 | | 99 |
|  | 35 | 20 | | 80 |
|  | 36 | 100 | | 0 |
|  | 40 | 100 | | 0 |

Table S3. Combination index (CI) of 18KHT01 for antioxidant activity

| **18KHT01 Concentration (µg/mL)** | **DPPH antioxidant activity** | | | **ABTS antioxidant activity** | | |
| --- | --- | --- | --- | --- | --- | --- |
|  | **DPPH radical scavenging (%)** | **CI** | **Combin-**  **ation effect** | **ABTS radical scavenging (%)** | **CI** | **Combin-**  **ation effect** |
| 125.0 | 97.27 | 0.54 | S | - | - | - |
| 62.5 | 96.72 | 0.36 | S | 100.168 | 0.58 | S |
| 31.25 | 94.53 | 0.40 | S | 100.335 | 0.17 | S |
| 15.625 | 90.15 | 0.53 | S | 100.335 | 0.08 | S |
| 7.8125 | 85.78 | 0.51 | S | 84.570 | 1.45 | A |
| 3.90625 | 76.48 | 0.67 | S | 52.872 | 1.17 | A |
| 1.95313 | 60.62 | 1.09 | A | 33.249 | 0.78 | S |
| 0.97656 | - | - | - | 22.683 | 0.48 | S |

The combination indexes (CI) for antioxidant activity of 18KHT01 were analyzed using CompuSyn software. The quantitative level of drug-drug interactions can be estimated as additive effect (CI = 1), synergism (CI < 1), and antagonism (CI > 1). The combination effect of ingredients in 18KHT01 for different concentrations are indicated as ‘A’ = antagonism, and ‘S’ = Synergism. CS = *Camellia sinensis*; QA = *Quercus acutissima*; GT = *Geranium thunbergii*; Lemon = *Citrus limon*.

Table S4. The primer sequence used for real-time PCR

| **Genes** | **Primer sequence (5'-3')** |
| --- | --- |
| **mPPARγ** | F: GTG AAG CCC ATC GAG GAC A  R: TGG AGC ACC TTG GCG AAC A |
| **mC/EBPα** | F: GCG GGA ACG CAA CAA CAT C  R: GTC ACT GGT CAA CTC CAG CAC |
| **maP2** | F: AGG CTC ATA GCA CCC TCC TGT G  R: CAG GTT CCC ACA AAG GCA TCA C |
| **mLeptin** | F: GCC AGG CTG CCA GAA TTG  R: CTG CCC CCC AGT TTG ATG |
| **mSREBP 1c** | F: GGT TTT GAA CGA CAT CGA AGA  R: CGG GAA GTC ACT GTC TTG GT |
| **mLPL** | F: TGT AAC AAT CTG GGC TAT GAG ATC AAC  R: TGC TTG CCA TCC TCA GTC CC |
| **mFAS** | F: GAA GCT CGT GTT GAC TTC TC  R: AGA AGA CCA CAA AGT AGT CCA G |
| **mβ-actin** | F: GTG ACG TTG ACA TCC GTA AAG A  R: GCC GGA CTC ATC GTA CTC C |

Table S5. Effect of *Q. acutissima* (QA), *G. thunbergii* (GT), *C. sinensis* (CS) and *C. limon* (Lemon) on cell viability of 3T3-L1 adipocytes

| **18KHT01 ingredients/groups** | **Treated concentrations** | **Cell viability (%)** |
| --- | --- | --- |
| Control | Blank | 100.00 ± 0.63 |
| High control | 1% Triton X | 0.00 ± 1.63 |
| *Quercus acutissima* (QA) | 80 µg/mL | 94.13 ± 12.64 |
| *Geranium thunbergii* (GT) | 80 µg/mL | 99.26 ± 2.04 |
| *Camellia sinensis* (CS) | 80 µg/mL | 100.74 ± 5.07 |
| *Citrus limon* (Lemon) | 80 µg/mL | 114.79 ± 1.69** |

The percentage of cell viability of samples was calculated as: cell viability (%) = 100-[ (Abs_sample_ – Abs_low control_)/( Abs_high control_ – Abs_low control_) × 100]. Statistical significance was calculated using one-way ANOVA followed by Dunnett's multiple comparisons test. Data are presented as as mean ± SD (n=6) with significance *******P* < 0.01 vs. control.

Table S6. Composition of experimental diets

| **Composition of standard chow diets** | | **Composition of high-fat diets** | |
| --- | --- | --- | --- |
| **Ingredients** | **Composition (%)** | **Ingredients** | **Composition (g/kg)** |
| Crude fiber | 6.7 | Casein | 200.0 |
| Amino acids | 18.4 | l-Cystine | 3.0 |
| Cholesterol (ppm) | 103 | Maltodextrin 10 | 125.0 |
| Total saturated fatty acids | 1.7 | Sucrose | 68.8.0 |
| Total monosaturated fatty acids | 1.4 | Lard | 245.0 |
| Starch | 33.9 | Soybean oil | 25.0 |
| Glucose | 0.3 | Cellulose BW200 | 50.0 |
| Fructose | 0.3 | Mineral mix S10026 | 10.0 |
| Sucrose | 2.0 | Calcium carbonate | 5.5 |
| Lactose | 0.3 | Potassium citrate | 16.5 |
| Calcium | 0.85 | DiCalcium Phosphate | 13.0 |
| Phosphorus | 0.62 | Vitamin mix V10001 | 10.0 |
| **Nutritional facts: % g (w/w) (% kcal)** | | | |
| **Protein** | 18.0 (20.1) | **Protein** | 26.2 (20.0) |
| **Carbohydrate** | 62.7 (65.30) | **Carbohydrate** | 26.3 (20.0) |
| **Fat** | 5.2 (13.67) | **Fat** | 34.9 (60.0) |

Table S7. Effect of 18KHT01 on Atherogenic index and percentage of protection

| **Groups** | **Atherogenic index (AI)** | **Protection (%)** |
| --- | --- | --- |
| Normal | 0.61 ± 0.18 | - |
| HFD | 1.43 ± 0.49^##^ | - |
| 18KHT01-75 | 1.26 ± 0.60^#^ | 11.75 |
| 18KHT01-150 | 0.45 ± 0.42** | 68.43 |
| Orlistat | 0.81 ± 0.08* | 43.37 |

Atherogenic index (AI) was calculated as follows: AI = (Total cholesterol – HDL)/HDL; and percentage of protection was calculated as: Protection (%) = (AI of HFD control – AI of treatment group)/AI of HFD control × 100. Statistical significance was calculated using one-way ANOVA followed by Dunnett's multiple comparisons test. Results are presented as the mean ± standard deviation (n=6) with significance #P < 0.05, ##P < 0.01 vs. Normal; *P < 0.05, **P < 0.01 vs. HFD control. Normal = Standard chow diet; HFD = high-fat diet control; 18KHT01-75 = 18KHT01 (75 mg/kg); 18KHT01-150 = 18KHT01 (150 mg/kg); Orlistat = orlistat (10 mg/kg).

Table S8. Effect of 18KHT01 on number and size of adipocytes on white adipose tissue

| **Groups** | **Feret’s diameter**  **(µm)** | **Mean area**  **(µm2)** | **Feret’s diameter**  **(µm)** |
| --- | --- | --- | --- |
| Normal | 542.50 ± 92.50 | 995.23 ± 323.13 | 46.78 ± 7.95 |
| HFD | 121.33 ± 19.82^###^ | 6204.16 ± 3345.96^###^ | 119.30 ± 25.96^###^ |
| 18KHT01-75 | 144.67 ± 25.79^###^ | 3957.64 ± 1579.30***^###^ | 97.36 ± 19.77***^###^ |
| 18KHT01-150 | 194.50 ± 30.96*^###^ | 2923.26 ± 885.07***^###^ | 79.79 ± 12.02***^###^ |
| Orlistat | 135.67 ± 11.13^###^ | 3849.57 ± 1169.24***^###^ | 95.89 ± 14.56***^###^ |

The images of WATs from H&E staining (20X magnification) were analyzed and adipocyte numbers, areas, and Feret’s diameters were evaluated using ImageJ software. Statistical significance was calculated using one-way ANOVA followed by Dunnett's multiple comparisons test. Results are presented as the mean ± standard deviation (n=6) with significance ###P < 0.001 vs. Normal; *P < 0.05, ***P < 0.001 vs. HFD control. Normal = Standard chow diet; HFD = high-fat diet control; 18KHT01-75 = 18KHT01 (75 mg/kg); 18KHT01-150 = 18KHT01 (150 mg/kg); Orlistat = orlistat (10 mg/kg).

**Table S9. Effect of 18KHT01 and its ingredients on weights of white adipose tissue, and liver**

| **Groups** | **WAT (g)** | **Liver (g)** |
| --- | --- | --- |
| Normal | 0.37 ± 0.07 | 0.75 ± 0.11 |
| HFD Control | 2.12 ± 0.76^###^ | 0.99 ± 0.05^###^ |
| 18KHT01 | 0.89 ± 0.25*** | 0.79 ± 0.08** |
| *C. sinensis* (CS) | 1.14 ± 0.34**^#^ | 0.79 ± 0.09** |
| *Q. acutissima* (QA) | 1.22 ± 0.39*^#^ | 0.72 ± 0.08*** |
| *G. thunbergii* (GT) | 2.40 ± 0.25 | 0.85 ± 0.02 |
| Orlistat | 1.48 ± 0.38^##^ | 0.81 ± 0.07** |

The visceral organs were isolated from each experimental groups and organ weight measured in situ. Statistical significance was calculated using one-way ANOVA followed by Dunnett’s multiple comparison test. Values are presented as the mean ± SD (n=5) with significance #P < 0.05, ##P < 0.01, ###P < 0.001 vs. Normal; *P < 0.05, **P < 0.01, ***P < 0.001 vs. HFD group. Normal = standard diet; HFD = high-fat diet control; CS = *Camellia sinensis* (150 mg/kg); QA = *Quercus acutissima* (150 mg/kg); GT = *Geranium thunbergii* (150 mg/kg); Orlistat = orlistat (10 mg/kg).

Table S10. Summary of clinical observations

| **Experiment days** | **Observations** | ***Q. acutissima***  **(150 mg/kg)** | ***G. thunbergii***  **(150 mg/kg)** | | |
| --- | --- | --- | --- | --- | --- |
|  |  | **Mice 1** | **Mice 1** | **Mice 2** | **Mice 3** |
| **Day 11** | 1st observation | ̶ | - Drowsiness | ̶ | ̶ |
|  | 2nd observation | ̶ | - Sedation,  - Laborious breathing,  - Drowsiness  - Piloerection | **-** Drowsiness | ̶ |
| **Day 12** | 1st observation | ̶ | **Dead** | - Sedation  - Laborious breathing  - Piloerection. | ̶ |
|  | 2nd observation | - Piloerection  - Hypoactive |  | More Severe toxicity. **Euthanized.** | ̶ |
| **Day 13** | 1st observation | - Sedation  - Laborious breathing |  |  | ̶ |
|  | 2nd observation | **Dead** |  |  | ̶ |
| **Day 18** | 1st observation |  |  |  | Mild toxic symptoms:  - Piloerection  - Hypoactive |
|  | 2nd observation |  |  |  | Mild toxic symptoms |
| **Day 20** | 1st observation |  |  |  | Mild toxic symptoms |
|  | 2nd observation |  |  |  | Severe toxic symptoms:  - Sedation  - Laborious breathing |
| **Day 21** | 1st observation |  |  |  | **Dead** |

̶ : absent of the clinical symptoms

**Table S11. Effect of 18KHT01 and its ingredients on liver and kidney-function parameters**

| **Groups** | **Creatinine (mg/dL)** | **Total bilirubin (mg/dL)** | **AST Activity (U/L)** | **ALT activity (U/L)** |
| --- | --- | --- | --- | --- |
| Normal | 1.72 ± 0.25 | 0.69 ± 0.13 | 20.10 ± 1.75 | 2.87 ± 0.36 |
| HFD Control | 1.68 ± 0.16 | 0.70 ± 0.11 | 27.19 ± 3.74^##^ | 4.58 ± 0.94^###^ |
| 18KHT01 | 1.60 ± 0.11 | 0.50 ± 0.13*^#^ | 24.78 ± 3.11 | 3.76 ± 0.61 ^#^ |
| *C. sinensis* (CS) | 1.73 ± 0.19 | 0.55 ± 0.09 | 24.76 ± 4.80 | 3.80 ± 0.28 ^#^ |
| *Q. acutissima* (QA) | 1.43 ± 0.12 | 0.59 ± 0.11 | 22.27 ± 1.98 | 3.93 ± 0.16 ^#^ |
| *G. thunbergii* (GT) | 1.61 ± 0.05 | 0.60 ± 0.03 | 39.23 ± 2.75 | 5.96 ± 0.57 |
| Orlistat | 1.54 ± 0.16 | 0.67 ± 0.06 | 21.78 ± 2.86* | 3.86 ± 0.28 ^#^ |

The levels of creatinine, total bilirubin and aspartate minotransferase (AST), and alanine aminotransferase (ALT) activities were determined in serum sample after nine weeks mice experiment. Statistical significance was calculated using one-way ANOVA followed by Dunnett's multiple comparisons test. Values are presented as the mean ± SD (n=5) with significance #P < 0.05, ##P < 0.01, ###P < 0.001 vs. Normal; *P < 0.05 vs. HFD group. Normal = standard diet; HFD = high-fat diet control; CS = *Camellia sinensis* (150 mg/kg); QA = *Quercus acutissima* (150 mg/kg); GT = *Geranium thunbergii* (150 mg/kg); Orlistat = orlistat (10 mg/kg).

Table S12. Effect of 18KHT01 and its ingredients on blood parameters

| **CBC parameters** | **Normal** | **HFD Control** | **18KHT01** | **CS** | **QA** | **GT** | **Orlistat** |
| --- | --- | --- | --- | --- | --- | --- | --- |
| **WBC**  **(x 10^9^/L)** | 2.72 ±0.6 | 3.30 ±0.6 | 2.53 ±0.3 | 2.33 ±0.3 | 2.28 ±1.2 | 2.80 ±1.7 | 2.88 ±1.1 |
| **LYM**  **(%)** | 76.10 ±4.9 | 74.80 ±2.7 | 75.27 ±3.6 | 76.83 ±2.8 | 76.63 ±1.3 | 80.30 ±0.1 | 77.83 ±4.5 |
| **MID**  **(%)** | 5.40 ±0.5 | 5.12 ±0.8 | 6.18 ±0.9 | 5.12 ±0.9 | 6.20 ±0.5 | 5.70 ±0.1 | 5.55 ±0.6 |
| **NEUT**  **(%)** | 18.50 ±4.6 | 20.08 ±3.0 | 18.55 ±3.0 | 18.05 ±2.5 | 17.18 ±0.8 | 13.85 ±0.2 | 16.62 ±4.7 |
| **LYM**  **(x 10^9^/L)** | 2.04 ±0.4 | 2.46 ±0.5 | 1.92 ±0.3 | 1.78 ±0.2 | 1.73 ±0.9 | 2.25 ±1.3 | 2.27 ±1.0 |
| **MID**  **(x 10^9^/L)** | 0.14 ±0.1 | 0.16 ±0.1 | 0.17 ±0.1 | 0.12 ±0.0 | 0.15 ±0.1 | 0.15 ±0.1 | 0.17 ±0.1 |
| **NEUT**  **(x 10^9^/L)** | 0.54 ±0.2 | 0.68 ±0.1 | 0.55 ±0.1 | 0.43 ±0.1** | 0.40 ±0.2** | 0.40 ±0.3 | 0.45 ±0.1* |
| **RBC**  **(x 10^12^/L)** | 6.19 ±0.4 | 6.61 ±0.1 | 6.46 ±0.5 | 5.99 ±0.4 | 5.78 ±0.7 | 6.12 ±1.7 | 6.52 ±0.6 |
| **HGB**  **(g/dL)** | 12.96 ±1.2 | 13.18 ±0.6 | 12.48 ±1.1 | 11.45 ±0.6* | 11.15 ±1.1* | 11.40 ±4.5 | 12.43 ±1.3 |
| **HCT**  **(%)** | 29.88 ±2.0 | 32.76 ±0.6 | 31.93 ±2.7 | 29.85 ±1.6 | 29.23 ±3.7 | 31.35 ±7.4 | 33.08 ±3.5 |
| **MCV**  **(fL)** | 48.30 ±0.3 | 49.66 ±0.4^#^ | 49.52 ±0.6^#^ | 49.95 ±0.7^##^ | 50.58 ±0.9^###^ | 51.60 ±2.0 | 50.72 ±1.0^###^ |
| **MCH**  **(pg)** | 20.86 ±0.8 | 19.90 ±0.6^#^ | 19.25 ±0.5^###^ | 19.00 ±0.4*^###^ | 19.25 ±0.4^###^ | 18.25 ±2.5 | 19.02 ±0.4^###^ |
| **MCHC**  **(g/dL)** | 43.28 ±1.8 | 40.16 ±1.4^##^ | 39.03 ±1.3^###^ | 38.33 ±0.6^###^ | 38.20 ±0.9^###^ | 35.60 ±5.9 | 37.55 ±0.9**^###^ |
| **RDW-SD**  **(fL)** | 20.78 ±0.9 | 21.92 ±0.9 | 21.03 ±1.0 | 23.20 ±1.0^##^ | 23.18 ±1.9^#^ | 26.00 ±5.2 | 23.50 ±0.9^##^ |
| **RDW-CV**  **(%)** | 12.62 ±0.6 | 12.90 ±0.4 | 12.45 ±0.5 | 13.60 ±0.4^#^ | 13.43 ±0.9 | 14.70 ±2.4 | 13.58 ±0.4^#^ |
| **PLT**  **(x 10^9/L)** | 398.20 ±93.2 | 422.00 ±24.0 | 436.83 ±63.5 | 489.50 ±35.5 | 759.50 ±132.2***^###^ | 913.50 ±58.7 | 619.00 ±52.0***^###^ |
| **MPV**  **(fL)** | 7.08 ±0.3 | 7.20 ±0.2 | 7.18 ±0.2 | 7.27 ±0.2 | 8.05 ±0.5***^###^ | 8.00 ±0.6 | 7.40 ±0.3 |
| **PDW**  **(%)** | 6.46 ±0.5 | 6.62 ±0.5 | 6.97 ±0.6 | 6.93 ±0.5 | 8.05 ±0.8**^###^ | 8.55 ±0.2 | 6.98 ±0.5 |

The blood samples were collected from each experimental groups and complete blood count was performed after nine weeks mice experiment. Statistical significance was calculated using one-way ANOVA followed by Dunnett's multiple comparisons test. Values are presented as the mean ± SD (n=5) with significance #P < 0.05, ##P < 0.01, ###P < 0.001 vs. Normal; *P < 0.05, **P < 0.01, ***P<0.001 vs. HFD group. Normal = standard diet; HFD = high-fat diet control; CS = *Camellia sinensis* (150 mg/kg); QA = *Quercus acutissima* (150 mg/kg); GT = *Geranium thunbergii* (150 mg/kg); Orlistat = orlistat (10 mg/kg).
